# Optimization of Pyrazole Compounds as Antibiotic Adjuvants Active against Colistin- and Carbapenem-Resistant *Acinetobacter baumannii*

**DOI:** 10.3390/antibiotics11121832

**Published:** 2022-12-16

**Authors:** Filomena Sannio, Antonella Brizzi, Rosita Del Prete, Marialuce Avigliano, Tiziana Simone, Carlotta Pagli, Teresa Ferraro, Filomena De Luca, Marco Paolino, Federico Corelli, Claudia Mugnaini, Jean-Denis Docquier

**Affiliations:** 1 Dipartimento di Biotecnologie Mediche, Università degli Studi di Siena, Viale Bracci 16, 53100 Siena, Italy; 2 Dipartimento di Biotecnologie, Chimica e Farmacia, Università degli Studi di Siena, Via Aldo Moro 2, 53100 Siena, Italy; 3 Lead Discovery Siena S.r.l., Via Aldo Moro 2, 53100 Siena, Italy; 4 Centre d’Ingénierie des Protéines—InBioS, Université de Liège, Allée du six Août 11, 4000 Liège, Belgium

**Keywords:** antibacterials, antibiotic adjuvant, colistin, ESKAPE bacteria, antibiotic potentiation, *Acinetobacter baumannii*

## Abstract

The diffusion of antibiotic-resistant, Gram-negative, opportunistic pathogens, an increasingly important global public health issue, causes a significant socioeconomic burden. *Acinetobacter baumannii* isolates, despite causing a lower number of infections than Enterobacterales, often show multidrug-resistant phenotypes. Carbapenem resistance is also rather common, prompting the WHO to include carbapenem-resistant *A. baumannii* as a “critical priority” for the discovery and development of new antibacterial agents. In a previous work, we identified several series of compounds showing either direct-acting or synergistic activity against relevant Gram-negative species, including *A. baumannii.* Among these, two pyrazole compounds, despite being devoid of any direct-acting activity, showed remarkable synergistic activity in the presence of a subinhibitory concentration of colistin on *K. pneumoniae* and *A. baumannii* and served as a starting point for the synthesis of new analogues. In this work, a new series of 47 pyrazole compounds was synthesized. Some compounds showed significant direct-acting antibacterial activity on Gram-positive organisms. Furthermore, an evaluation of their activity as potential antibiotic adjuvants allowed for the identification of two highly active compounds on MDR *Acinetobacter baumannii*, including colistin-resistant isolates. This work confirms the interest in pyrazole amides as a starting point for the optimization of synergistic antibacterial compounds active on antibiotic-resistant, Gram-negative pathogens.

## 1. Introduction

Antibacterial agents of both natural and synthetic origins represent invaluable therapeutic resources that not only have allowed for the effective treatment of once deadly bacterial infections, but also have paved the way for (and still is a pillar of) modern medicine (including organ transplantations, invasive surgery, etc.). However, the effectiveness of antibacterial drugs is undermined by the emergence and spread of resistant strains, which have developed resistance mechanisms to evade the activity of virtually all classes of antibacterial drugs [1,2]. Several other factors (the low financial attractiveness of antibiotics and costs of discovery and development, the evolution of clinically relevant species with multidrug resistance, and more stringent requirements for the approval of new drugs) have led to a significant shortage of new antibacterial drugs on the market in the last two decades and, consequently, to the increase in resistance to antibiotics, especially carbapenems, which are increasingly used to treat infections caused by multidrug-resistant (MDR) bacteria [3]. Inevitably, the current crisis due to antibiotic resistance is characterized by a significant increase in the prevalence of carbapenem-resistant, Gram-negative, opportunistic pathogens (Enterobacterales, *Pseudomonas* spp., and *Acinetobacter* spp.), which have caused a large number of deaths and comorbidities all around the world and have been identified by the WHO as critical priorities for the development of new antibiotics [4]. Furthermore, the recent increase in the prevalence of colistin resistance (due to a variety of mechanisms depending on the host) has allowed relevant Gram-negative bacteria to evolve towards ultra-resistant phenotypes, and consequently, has further narrowed our therapeutic options.

*Acinetobacter baumannii* (Aba) is an emblematic example of this as it has rapidly evolved into having ultra-resistant phenotypes, especially in relation to the significant increase, over the last decade, in the use of last-resort drugs. Commonly resistant to colistin [5] and carbapenems (due to the production of a variety of carbapenemases), Aba isolates are also refractory to the more recent combinations of β-lactams with diazabicyclooctanone-based (DBO; ceftazidime/avibactam and imipenem/cilastatin/relebactam) or boronate-based (meropenem/vaborbactam) β-lactamase inhibitors [6,7]. Furthermore, only a few compounds show a spectrum of activity that includes this organism (eravacycline, approved in September 2018; and, to some extent, cefiderocol, approved in April 2020), and therapeutic solutions targeting extensively drug-resistant (XDR) Aba that is resistant to carbapenems and colistin (sulbactam/durlobactam and BV100, phase 1; meropenem/ANT3310, preclinical phase) are in the early stages of development [8]. There is still an urgent need to explore alternative strategies for treating infections caused by ultra-resistant Aba isolates.

One of the fastest ways to obtain valuable antibacterial drugs active against resistant bacteria and susceptible to clinical development is based on: (1) combinations of compounds that exhibit “antibiotic enhancer” [9] or “resistance breaker” [10] activity with available antibiotics and (2) the use of new technological platforms for the formulation of last-resort antibiotics (e.g., conjugated with antibodies or nanoparticles [11]). Other innovative approaches include the design of antivirulence compounds, e.g., by targeting important bacterial regulators of biofilm formation or virulence, or by directly inhibiting virulence factors.

Following an extensive screening campaign for the identification of synthetic compounds with direct or synergistic antibacterial activity, we recently identified a number of compounds showing synergistic activity with colistin on various Gram-negative pathogens, including *K. pneumoniae*, *P. aeruginosa*, and Aba [12]. Most of the compounds identified belong to two structural classes (substituted phenols and pyrazoles) that could serve as a basis for the optimization of colistin resistance breakers and could be used in combination for the treatment of multidrug-resistant Aba infections.

**Figure 1:**
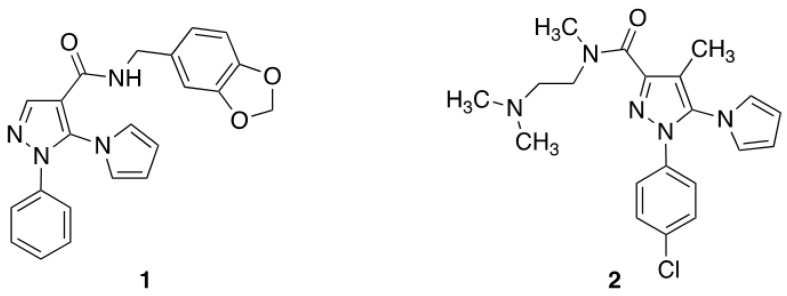
Chemical structure of hit compounds identified during a previous antibacterial screening campaign of an unfocused library [12].

In view of the potentially problematic nature of phenol derivatives from the pharmacokinetic point of view, we have given priority to the pyrazole scaffold and selected compounds **1** and **2** (Figure 1) as a starting point for optimization, with the aim of identifying more active compounds as colistin resistance breakers acting on MDR Aba isolates.

## 2. Results

### 2.1. Chemistry

**Figure 2:**
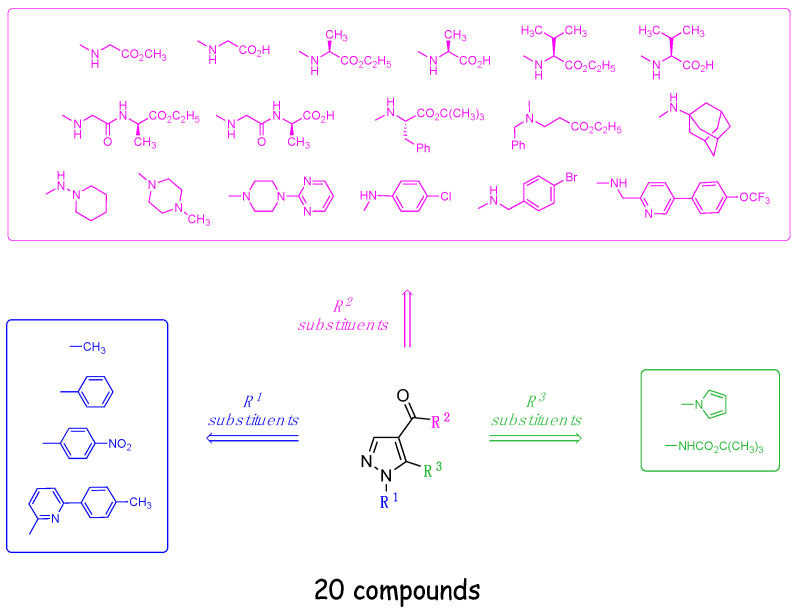
Exploration of the chemical space around pyrazole-4-carboxamides.

**Figure 3:**
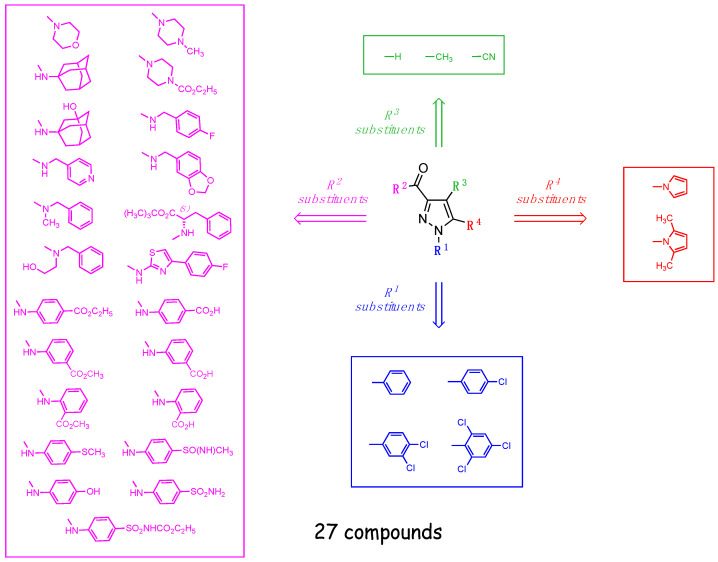
Exploration of the chemical space around pyrazole-3-carboxamides.

**Scheme 1:**
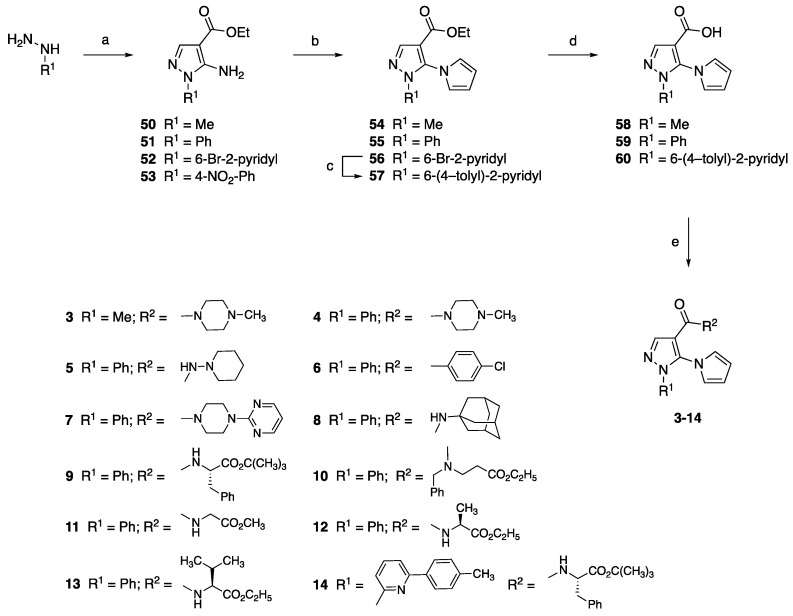
Synthesis of pyrazole-4-carboxamide derivatives **3**–**14**. Reagents and conditions: (a) diethyl ethoxymethylenemalonate, EtOH, reflux; (b) 2,5-dimethoxytetrahydrofuran, glacial acetic acid, 120 °C; (c) p-tolylboronic acid, Pd(OAc)_2_, PPh_3_, 1 M Na_2_CO_3_, dioxane, reflux, N_2_ atmosphere, 4 h; (d) NaOH, H_2_O, reflux; (e) appropriate amine, HOBt, EDC, DCM, RT.

**Scheme 2:**
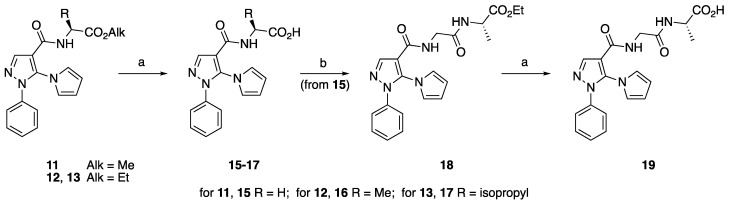
Synthesis of pyrazole-4-carboxamide derivatives **15**–**19**. Reagents and conditions: (a) LiOH, H_2_O/THF/MeOH, RT, 4 h; (b) (R)-alanine ethyl ester hydrochloride or (R)-valine ethyl ester hydrochloride, HOBt, EDC, DCM, RT.

**Scheme 3:**
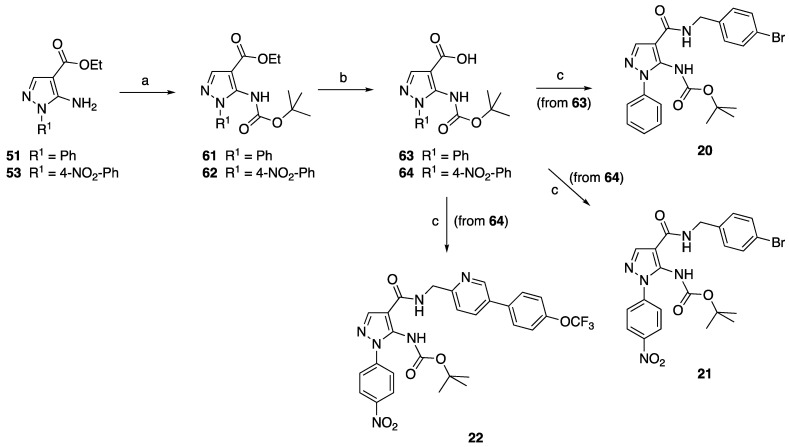
Synthesis of pyrazole-4-carboxamide derivatives **20**–**22**. Reagents and conditions: (a) Boc_2_O, DCM, RT, 2–4 h; (b) KOH, EtOH/THF, 55 °C, 1 h; (c) appropriate amine, HOBt, EDC, DCM, RT.

**Scheme 4:**
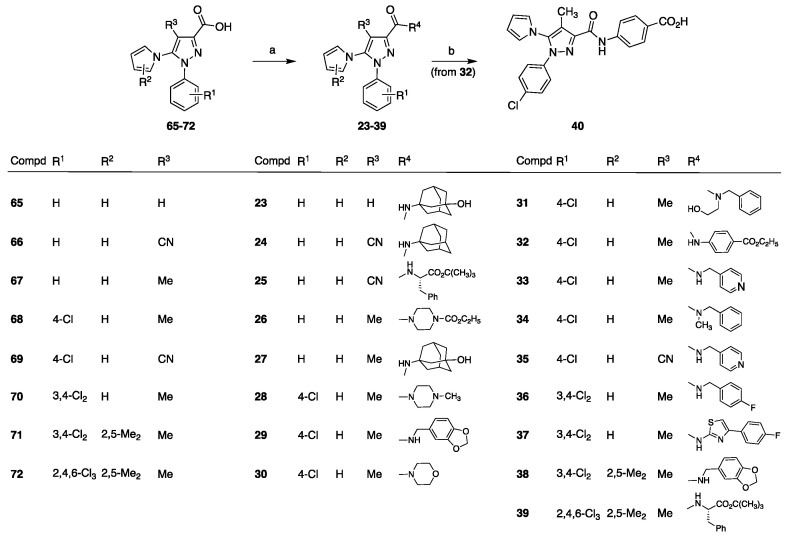
Synthesis of pyrazole-3-carboxamide derivatives **23**–**40**. Reagents and conditions: (a) appropriate amine, HOBt, EDC, DCM, RT; (b) NaOH, MeOH, H_2_O, reflux, 3 h.

**Scheme 5:**
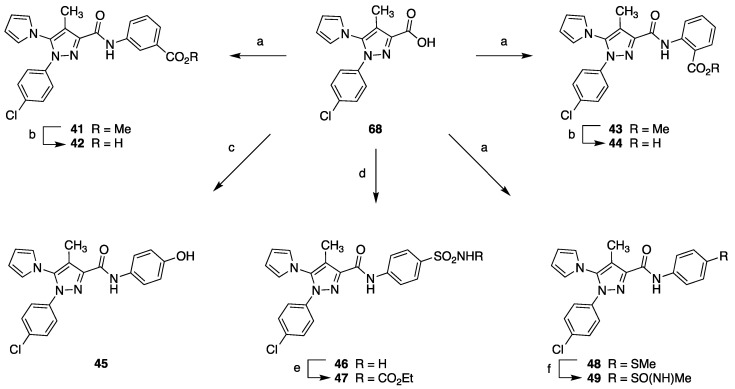
Synthesis of pyrazole-3-carboxamide derivatives **41**–**49**. Reagents and conditions: (a) appropriate aniline, HOBt, EDC, DCM, RT; (b) NaOH, MeOH, H_2_O, reflux, 3 h; (c) 4-aminophenol, PyBOP, HOBt, dry DMF, RT, 4 h; (d) (COCl)_2_, DCM, RT, 1 h, and then sulfanilamide, TEA, dry THF, RT, 2 h; (e) methyl chloroformate, TEA, DCM, RT, 1 h; (f) PhI(OAc)_2_, NH_4_OH, MeOH, RT, 2 h.

Considering the paucity of information available regarding the structural determinants for synergistic antibacterial activity with the colistin of hit compounds 1 and 2, we kept the pyrazole ring as the critical structural element and undertook a wide exploration of the chemical space around this ring to obtain, in the end, a library of 47 pyrazole derivatives. Based on the hit compound **1**, a first group of 20 pyrazole 4-carboxamide derivatives (Figure 2) was synthesized by varying substituents R1, R2, and R3, whereas a wider investigation of chemical substitution at every position of the pyrazole ring, starting from the hit compound **2**, led to a second family of derivatives characterized by the presence of a carboxamide substituent at position 3 (Figure 3). No particular attention was paid to the design of compounds with optimal physicochemical properties, favoring, in the first instance, the exploration of chemical space, and postponing the structural optimization of the most promising compounds to a later stage. Compounds **3**–**22**, featuring a carboxamide group at position 4, were prepared as outlined in Scheme 1, Scheme 2 and Scheme 3, whereas the preparation of compounds **23**–**49**, featuring a carboxamide substituent at position 3 of the pyrazole nucleus, as shown by the hit compound **2**, is described in Scheme 4 and Scheme 5. The reaction between the appropriate hydrazines and diethyl ethoxymethylenemalonate (Scheme 1) yielded the 5-aminopyrazole derivatives **50**–**53**, of which **50**–**52** were converted into the pyrrol-1-yl derivatives **54**–**56** by using the Clauson-Kaas procedure [13]. Compound **56** was subjected to a Suzuki–Miyaura reaction with p-tolylboronic acid to produce compound **57**. Esters **54**, **55**, and **57** were, in turn, hydrolyzed [14] to the corresponding acids **58**–**60**, which were subjected to an amidation reaction, which was conducted by following previously published procedures [15], to give the final amides **3**–**14**. 

Alkyl esters **11**–**13** (Scheme 2) were further elaborated by using hydrolysis on the corresponding acids **15**–**17**, and acid **15** was reacted with L-alanine ethyl ester to yield **18**, which, in turn, was converted into acid **19**. Ethyl 5-amino-1H-pyrazole-4-carboxylates **51** and **53** (Scheme 3) were acylated with di-tert-butyl dicarbonate to give the N-Boc derivatives **61** and **62**, which were, subsequently, hydrolyzed to carboxylic acids **63** and **64**, respectively. Amidation of these carboxylic acids using commercially available 4-bromobenzylamine or (5-(4-(trifluoromethoxy)phenyl)pyridine-2-yl)methanamine [16] led to the final compounds **20**–**22**.

Compounds **23**–**39** (Scheme 4) were synthesized through a condensation reaction with the appropriate amines under usual conditions starting from the carboxylic acids **65**–**72**, which were prepared as previously reported [15]. Ester **32** was then hydrolyzed to obtain the corresponding acid **40**.

To investigate whether the particular antibacterial properties of compound **40** (vide infra) might depend on the acidity of its carboxyl group or on the position of the same on the benzene ring, a small family of compounds was synthesized (Scheme 5) in which the carboxyl group was shifted to the meta- or ortho-position (compounds **42** and **44**), or replaced by other functional groups with different p*K_a_* values, namely, a phenolic hydroxyl (**45**) or a sulfonamide (**46**), acylsulfonamide (**47**), or sulfoximine group (**49**). Acid **68** was amidated with methyl 3-aminobenzoate using EDC/HOBt as coupling reagents to give the intermediate **41**, which was then hydrolyzed (NaOH/MeOH) to obtain the free carboxylic acid **42**. Similarly, **68** was also converted to **43** using 2-aminobenzoate methyl ester, which was then hydrolyzed to **44**, whereas for the preparation of **45**, 4-aminophenol PyBOP/HOBt in dry DMF was used. The activation of **68** to the corresponding acyl chloride followed by a reaction with sulfanilamide led to **46**, which was, subsequently, transformed into the acylsulfonamide **47** via a treatment with methyl chloroformate. The reaction between acid **68** and 4-methylthioaniline, as described for the synthesis of **41** and **43**, gave the amide **48** which, when treated with (diacetoxyiodo)benzene in the presence of ammonium hydroxide as a nitrogen source, gave the sulfoximine derivative **49**.

### 2.2. Biological Evaluation of Pyrazole-4- and Pyrazole-3-Carboxamide Derivatives

**Figure 4:**
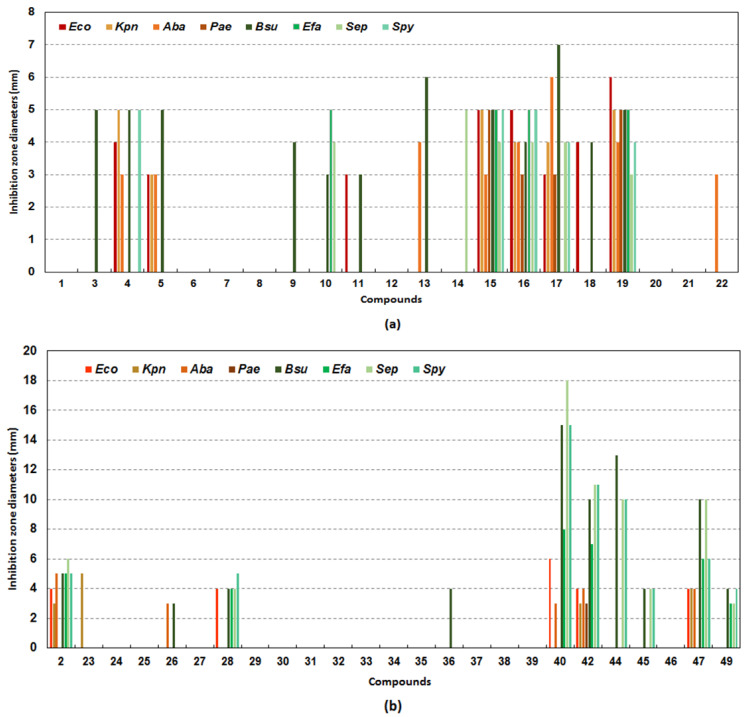
Direct antibacterial activity of compounds **3**–**40**, **42**, **44**–**47**, and **49** (agar diffusion method) on a panel of eight representative bacterial species (type strains), including four Gram-negative and four Gram-positive organisms. (**a**) Pyrazole-4-carboxamide analogues (data for parent compound **1** are shown for comparison); (**b**) pyrazole-3-carboxamide analogues (data for parent compound **2** are shown for comparison) [12].

Compounds **3**–**40**, **42**, **44**–**47**, and **49** (Scheme 1, Scheme 2, Scheme 3, Scheme 4 and Scheme 5) were subjected to a detailed investigation of their direct and synergistic antibacterial activity and potential cytotoxicity by using several methods. The direct antibacterial activity of these compounds was determined using both agar diffusion and broth microdilution methods. The former was initially used to identify potentially active compounds and was tested on eight reference strains, with representatives of both Gram-positive and Gram-negative clinically relevant bacterial species or genera (Figure 4).

As somewhat expected, considering that the parent compounds were selected for their synergistic rather than direct-acting antibacterial activity [12], limited growth inhibition was observed for these compounds as the best compounds showed a growth inhibition zone with a diameter of a max. of 7 mm. Interestingly, some compounds, specifically **15**, **16**, **17**, **19**, and **42**, showed a broad-spectrum, though moderate, direct-acting antibacterial activity. All these compounds, which are analogues of parent compound **1**, but where no direct antibacterial activity was observed [12], are characterized by the presence of free carboxylic acid on the carboxamide substituent at position 4 of the pyrazole heterocycle. However, none of these compounds exhibited MIC values lower than 512 µg/mL when tested using a broth microdilution method.

Notable exceptions were represented by **40**, **42**, **44**, and **47**, which showed a remarkable antibacterial activity on Gram-positive organisms (although slightly lower on *Enterococcus faecalis*), with growth inhibition zones having diameters of up to 18 mm. This activity was confirmed in broth microdilution assays and translated in MIC values ranging from 32 µg/mL for *Bacillus subtilis*, *Enterococcus faecalis*, and *Staphylococcus aureus*, to 16 µg/mL for *Streptococcus pyogenes*. From a structural standpoint, these four appear to be related to parent compound **2**, as they feature a 3-carboxamide (rather than a 4-carboxamide) substituent. Interestingly, the 3-carboxamide substituent also contains a free carboxylic group in three of these compounds, although the nature of the linker varies from that of the analogs of **1**, being made up of a phenyl rather than an aliphatic chain. Overall, these data suggest that the introduction of a free carboxyl group into parent compounds **1** and **2** leads to an improvement, albeit modest overall, in their direct-acting antibacterial activity against Gram-positive organisms, which is maintained when the acid group is moved to other positions on the benzene ring or replaced by a functional group, such an acylsulfonamide, with a similar p*K_a_* value. Although potentially interesting, none of these compounds exhibited sufficient activity on Gram-negative bacteria, which nowadays, represent a far more relevant target for the discovery of novel antibacterial scaffolds, and were not further studied in this work. 

Second, we established a rapid screening method to identify compounds showing synergistic activity with antibiotics on representative Gram-negative species. This method was based on the detection of growth inhibition in liquid medium (Mueller–Hinton II culture medium) in the presence of a single concentration of the compound (64 µg/mL) and subinhibitory concentrations (0.5 × MIC) of colistin (see the Materials and Methods section for details), as this antibiotic appears to be the best potentiator of parent compounds [12]. From this analysis, 11 compounds (**8**, **9**, **13**, **20**, **22**, **27**, **28**, **31**, **39**, **45**, and **49**) were identified that showed synergistic activity on at least one organism (out of the four representative Gram-negative bacteria) (Table 1). 

Interestingly, these compounds were structurally different from those showing direct-acting activity, and were analogues of both parent compounds **1** and **2**. Compound **39** showed synergistic activity with colistin on Aba only. A broader spectrum of activity was observed with all other compounds, most notably, **9**, **28**, **31**, **45**, and **49**, which inhibited the growth of all tested bacteria except *P. aeruginosa*. None of the compounds actually proved active on that organism. This synergistic activity was further investigated by determining the MIC values of these compounds in the presence of a subinhibitory concentration of colistin and provided additional data regarding their antibacterial spectrum and potency (Table 2).

**Figure 5:**
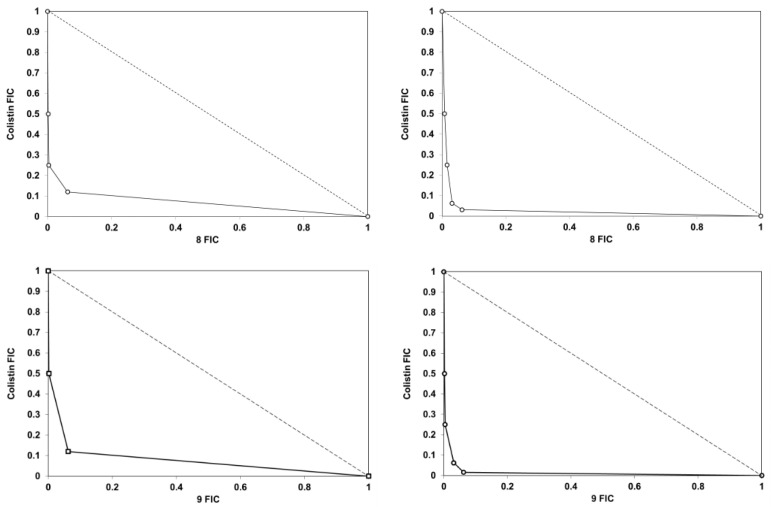
Isobolograms showing the strong synergistic activity of compounds **8** (**top**) and **9** (**bottom**) with colistin on *A. baumannii* reference strain (ATCC 17978) (**left**) and an MDR, colistin-resistant, and carbapenem-resistant *A. baumannii* clinical isolate (N50) (**right**).

Compounds **9**, **45**, and **49** emerged as the most promising pyrazolo-carboxamide derivatives, with MAC values ranging from 4 to 8 µg/mL on both Aba and Enterobacterales, interestingly showing that both scaffolds (pyrazole-4- and pyrazole-3-carboxamides) allowed for the identification of potent compounds showing synergistic activity with colistin. A similarly potent synergistic activity was also observed with compound **8** on Aba. Compounds **8** and **9** are analogues of parent compound **1**, with modifications of the nature of the 4-carboxamide substituent (e.g., adamantanyl). Other modifications of compound **1**, including the introduction of butanoate ester on the 4-carboxamide substituent (**13**) or the substitution of the 5-pyrrole ring with a *tert*-butoxycarbonylamino moiety (**20** and **22**), did not translate into a similar increase in antibacterial synergistic activity. Regarding pyrazole-3-carboxamide derivatives, some new analogues of compound **2** (**27**, **28**, **31**, and **39**) did not prove to be significantly better than the parent compound. However, compounds **45** and **49** did provide some form of significant improvement in their synergistic activity, when compared to that of parent compound **2**. Compounds **8**, **9**, **45**, and **49** showed the best overall activity, including on Aba, which we consider a more relevant bacterial target in our drug discovery programs than Enterobacterales, in relation to the paucity of antibiotics currently in clinical development targeting this specific pathogen (see Introduction), and were selected for further investigation. A chequerboard analysis, to further assess whether they would exhibit true synergistic activity or rather a simple additive effect with colistin, was carried out (Table 3, Figure 5). All new tested compounds (**8**, **9**, **45**, and **49**) were confirmed to be synergistic with colistin on antibiotic-susceptible reference strains of *E. coli*, *K. pneumoniae*, and Aba. Compound **8** was slightly inferior to **9**, as expected from the data reported in Table 2. A different picture emerged when these compounds were tested on clinical isolates showing a multidrug- or pandrug-resistant phenotype, including to polymyxin antibiotics. On one hand, synergistic activity could not be observed with the pan-resistant *K. pneumoniae* isolate, whose resistance to colistin was acquired through the insertional inactivation of *mgrB* (a negative regulator of PhoP/PhoQ) [17]. On the other hand, the potentiation effect was stronger on a colistin-resistant Aba strain when compared to that of the antibiotic-susceptible reference strains, as the resulting average FIC index values were lower when measured on the colistin-resistant Aba strain (Figure 5).

These data confirmed that the new analogues **8** and **9** were promising potentiators of polymyxin antibiotics, especially on colistin-resistant Aba. Encouraged by these results, we wanted to understand whether any of these two active compounds would show sufficient selectivity, and thus, validate the scaffold as a potentially useful new series of antibiotic adjuvants. The potential cytotoxic activity of both compounds was assessed using several methods. First, a simple membrane integrity assay carried out on HeLa cells showed that none of the compounds induced an LDH release at concentrations of up to 256 µg/mL after 24 h of exposure (Table 4). This is in agreement with the fact that no hemolytic activity could be observed, not only for compounds **8** and **9**, but also for compounds **1**, **2**, **45**, and **49**. Considering that these compounds would show potent antibacterial activity in the presence of colistin (a membrane-interacting antibiotic known for its suboptimal safety profile [19,20]), we also wanted to investigate whether the cytotoxicity of our compounds would be affected by the presence of this antibiotic. Colistin, when tested alone in a similar assay, showed an IC_50_ value of 1240 µg/mL, confirming its membrane-damaging activity in eukaryotic cells at higher concentrations. Based on this result, the cytotoxicity of our compounds was measured in the presence of a subtoxic concentration of colistin (512 µg/mL). In this case, compounds **8** and **9** showed a significantly different behavior, as compound **8** appeared to enhance the membrane-damaging activity of colistin at rather low concentrations (IC_50_, 16.4 µg/mL). Interestingly, this was not observed with compound **9**, which was devoid of any membrane-damaging activity both in the absence and in the presence of 512 µg/mL of colistin. Subsequently, the cytotoxicity of compound **9** was further assessed using cell proliferation/viability assays (see the Materials and \methods section for details). In these assays, the proliferation of HeLa cells was not significantly altered in the presence of 16 µg/mL of compound for up to 72 h. Moreover, similar results were obtained when this experiment was performed in the presence of 512 µg/mL of colistin in the medium.

**Figure 6:**
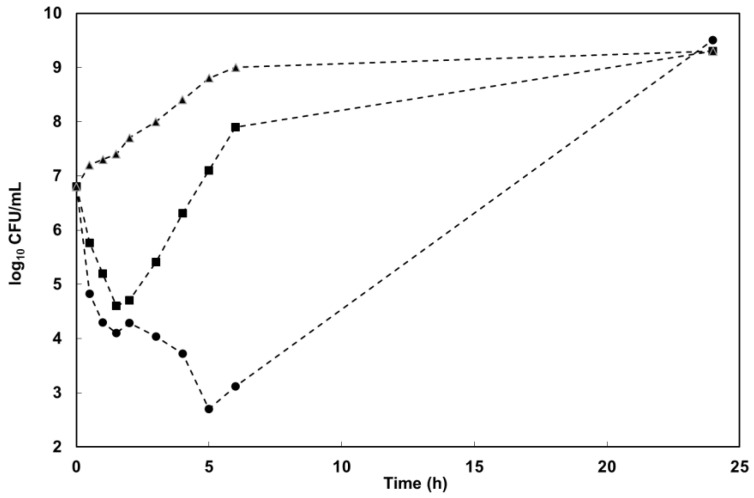
Time-dependent kill-curve analysis of the MDR, colistin-resistant *A. baumannii* clinical isolate (N50) in the presence 2 µg/mL of colistin (squares), 2 µg/mL of colistin, and 8 µg/mL of **9** (circles). The growth control is shown as triangles (similar results were obtained in the presence of 8 µg/mL of **9**, as this compound alone does not exhibit antibacterial activity).

Finally, and considering the promising selectivity showed by compound **9**, its antibacterial activity with colistin on the colistin-resistant Aba N50 strain was further characterized using time-kill curves (Figure 6). The presence of colistin alone (tested at 2 µg/mL, i.e., the susceptibility breakpoint), although showing an initial moderate impact on bacterial survival, was not expectedly able to show any bactericidal effect (defined as the capacity of a compound to decrease the initial bacterial population by at least 3 log_10_ [21]). However, a combination of 2 µg/mL of colistin and 8 µg/mL of **9** did exhibit a significantly faster and better bacterial killing, with a 4 log_10_ reduction achieved within 5 h. Unfortunately, complete eradication could not achieved, leading to the recovery of the bacterial growth. This was confirmed by the determination of the MBC, which yielded values >64 µg/mL for compounds **8**, **9**, **45**, and **49** when tested in the presence of 2 µg/mL of colistin.

## 3. Discussion and Conclusions

In this study, we investigated the direct-acting and synergistic antibacterial activity of 44 new analogues of pyrazole-3-carboxamides and pyrazole-4-carboxamides identified in a previous work [12]. A single compound showed significant direct-acting activity primarily on Gram-positive organisms, a result that was somewhat expected considering that the parent compounds were previously identified and characterized for their synergistic activity with colistin. Interestingly, nine new analogues of both parent compounds **1** and **2** maintained a synergistic activity on Gram-negative bacteria, with the exception of *P. aeruginosa*. Two compounds, **8** and **9**, were significantly more active than their parent compound **1**, and were characterized by a different substituent on the 4-carboxamide moiety. Apparently, this structural difference largely affects the properties of compound **8**, which not only exhibited a narrower spectrum of synergistic activity, but also a more apparent enhancement of the membrane-damaging activity of colistin on eukaryotic cells. More strikingly, compound **9** showed synergistic activity on both colistin-susceptible Enterobacterales and Aba, but also proved, in combination with a subinhibitory concentration of colistin, to significantly enhance the rate of killing of a colistin-resistant Aba clinical isolate at concentrations as low as 8 µg/mL while showing a promising selectivity. Furthermore, compounds **45** and **49**, which are pyrazole-3-carboxamide derivatives, also proved to have a better synergistic activity than their parent **2**, although the improvement on Aba was less remarkable. These data, overall, further highlight the potential of pyrazole-4- and pyrazolo-3-carboxamide analogues to obtain selective and synergistic compounds to be further optimized, and hopefully represent a promising source of new therapeutic solutions that are able to restore the activity of the last-resort polymyxins for the treatment of infections caused by extensively drug- or pandrug-resistant clinical isolates of *Acinetobacter baumannii*. Furthermore, the synergistic activity exhibited by these compounds in the presence of colistin may rely on the permeabilizing effect of the latter, and provides hope that the further optimization of these compounds, particularly regarding their rate of diffusion through bacterial membranes, may allow for the identification of potent and direct-acting compounds.

## 4. Materials and Methods

### 4.1. Chemistry

Reagents were purchased from commercial suppliers and used without further purification. Anhydrous reactions were run under a positive pressure of dry N_2_. Merck silica gel 60 was used for flash chromatography (23–400 mesh). ^1^H NMR and ^13^C NMR were recorded at 400 and 100 MHz, respectively, on a Bruker Advance DPX400. Chemical shifts are reported relative to tetramethylsilane at 0.00 ppm. Mass spectral (MS) data were obtained using the Agilent 1100 LC/MSD VL system (G1946C) with a 0.4 mL/min flow rate and using a binary solvent system of 95:5 methanol/water. UV detection was monitored at 254 nm. Mass spectra were acquired either in positive or in negative mode by scanning over the mass range of 105–1500. Melting points were determined on a Gallenkamp apparatus and are uncorrected. Elemental analyses were performed on a PerkinElmer PE 2004 elemental analyzer and the data for C, H, and N are within 0.4% of the theoretical values. The chemical purity of most of the target compounds was determined using an ACQUITY Waters UPLC-MS system with a Waters BEH C18 reversed-phase column (2.1 mm × 50 mm, 1.7 µm); the method was carried out under the following conditions: gradient elution, solvent A (0.1% formic acid in water), solvent B (0.1% formic acid in acetonitrile) 90:10 to 0:100 over 2.9 min, flow rate of 0.5 mL/min, UV detector, and 254 nm. The chemical purity of compounds **10**, **14**, and **19** was determined using an Agilent 1260 Infinity instrument constituted of a binary pump, an autosampler, an UV-DAD, and an ESI-MS detector. The chromatographic separation was realized with a Symmetry^®^ C18 column (4.6 × 75 mm, 3.5 µm). Analysis was carried out with methanol as the mobile phase at a flow rate of 0.5 mL/min. UV detection was monitored at 254 nm. The purity of each compound was ≥ 95% in either analysis, with the exception of compounds **3**, **4**, **6**, **7**, and **20**, whose purity was in the range of 90–94%; accordingly, elemental analyses were not performed on these compounds.

#### 4.1.1. Synthesis of Aminopyrazole Precursors **50**–**53**

Well-established procedures from the literature were applied for the synthesis of **50 [22]**, **51** [15], **52** [23], and **53** [24].

#### 4.1.2. General Procedure for the Synthesis of Ethyl 5-Pyrrol-1-yl-1*H*-pyrazole-4-carboxylates **54**–**56**

Compounds **54** and **55** were prepared according to known procedures [15,25]. As an example, the preparation of the new compound **56** is reported below.

##### Ethyl 5-Amino-1-(6-bromopyridin-2-yl)-1*H*-pyrazole-4-carboxylate (**56**)

A solution of AcONa (1.6 g, 11.7 mmol) in water (2.5 mL) was added to a solution of ethyl (ethoxymethylene)cyanoacetate (988 mg, 5.85 mmol) in glacial AcOH (15 mL). After stirring for a few minutes, 6-bromo-2-pyridinylhydrazine (1.0 g, 5.32 mmol) was added and the mixture was stirred at 100 °C overnight. After cooling, the solution was poured into ice water and the precipitate which formed was suction-filtered and washed thoroughly with PE. Flash chromatographic purification on a silica gel with DCM/PE (3:1) as the eluent produced the title compound **56** (76% yield) as a white solid. Mp 163–166 °C. ^1^H NMR (400 MHz, CDCl_3_): δ 7.85 (d, *J* = 8.2 Hz, 1H), 7.71 (s, 1H), 7.61 (t, *J* = 8.0 Hz, 1H), 7.27 (d, *J* = 7.7 Hz, 1H), 4.24 (q, *J* = 14.2, 7.1 Hz, 2H), 1.30 (t, *J* = 7.1 Hz, 3H). MS (ESI): *m*/*z* 311 [M + Na]^+^.

#### 4.1.3. Ethyl 5-(1*H*-Pyrrol-1-yl)-1-[6-(p-tolyl)pyridin-2-yl]-1*H*-pyrazole-4-carboxylate (**57**)

To a solution of **56** (100 mg, 0.27 mmol) in 1,4-dioxane (4 mL), Pd(OAc)_2_ (6.04 mg, 0.027 mmol), PPh_3_ (21.2 mg, 0.08 mmol), and p-tolylboronic acid (110 mg, 0.81 mmol) were added successively. After flushing with N_2_, a 1 M Na_2_CO_3_ solution (0.5 mL, 0.5 mmol) was added and the reaction mixture was refluxed for 4 h under N_2_. The solvent was removed under reduced pressure and the residue was taken up in EtOAc and filtered on celite. The solution was dried over anhydrous sodium sulfate and concentrated to leave a residue, which was purified through flash chromatography on a silica gel (PE/EtOAc, 3:1) to produce **57** (77% yield) as a white solid. Mp 124–126 °C. ^1^H NMR (400 MHz, CDCl_3_): δ 8.11 (s, 1H), 7.77 (t, *J* = 7.9 Hz, 1H), 7.61 (d, *J* = 7.8 Hz, 1H), 7.54 (d, *J* = 8.2 Hz, 2H), 7.37 (d, *J* = 7.9 Hz, 1H), 7.13 (d, *J* = 8.0 Hz, 2H), 6.80–6.72 (m, 2H), 6.26–6.24 (m, 2H), 4.19 (q, *J* = 14.2, 7.1 Hz, 2H), 2.31 (s, 3H), 1.20 (t, *J* = 7.1 Hz, 3H). MS (ESI): *m*/*z* 372 [M + H]^+^.

#### 4.1.4. General Procedure for the Synthesis of 5-(1*H*-Pyrrol-1-yl)-1*H*-pyrazole-4-carboxylic Acids **58**–**60**

Compounds **58** and **59** were prepared according to known procedures [15,25]. As an example, the preparation of the new compound **60** (5-(1*H*-Pyrrol-1-yl)-1-[6-(p-tolyl)pyridin-2-yl]-1*H*-pyrazole-4-carboxylic acid) is reported below.

A mixture of **57** (200 mg, 0.54 mmol) in EtOH (4 mL) and NaOH (216 mg, 5.4 mmol) in water (4 mL) was heated at reflux for 1 h, then cooled in an ice bath and acidified with conc. HCl. The precipitate was extracted with EtOAc and the combined organic layer was dried over sodium sulfate and evaporated under reduced pressure. The solid residue was triturated with PE/Et_2_O to give the title compound **60** (81% yield) as a white solid. Mp 240–243 °C. ^1^H NMR (400 MHz, CDCl_3_): δ 8.17 (s, 1H), 7.78 (t, *J* = 8.0 Hz, 1H), 7.63 (d, *J* = 7.9 Hz, 1H), 7.54 (d, *J* = 8.1 Hz, 2H), 7.35 (d, *J* = 7.9 Hz, 1H), 7.13 (d, *J* = 7.9 Hz, 2H), 6.77–6.75 (m, 2H), 6.26 (d, *J* = 2.0 Hz, 2H), 2.32 (s, 3H). MS (ESI): *m*/*z* 344 [M − H]^−^.

#### 4.1.5. General Procedure for the Synthesis of Amide Derivatives **3**–**14**

To a solution of the carboxylic acids **58**–**60** (1 mmol) in DCM (50 mL), HOBt (135 mg, 1 mmol), EDC (383 mg, 1.2 mmol), and the appropriate amine (1.5 mmol) were added successively. After stirring at room temperature for 12 h, the solution was washed with 1 N HCl, 10% NaHCO_3_ solution, and brine. After drying on sodium sulfate, the solvent was removed under reduced pressure and the residue was purified by recrystallization from MeOH or flash chromatography on a silica gel using the reported eluent system.

##### (4-Methylpiperazin-1-yl)[1-methyl-5-(1*H*-pyrrol-1-yl)-1*H*-pyrazol-4-yl]methanone (**3**)

Prepared from the acid **58** and 1-methylpiperazine. Eluent: DCM/MeOH (97:3). Yield: 68%. Mp 109–111 °C. ^1^H NMR (400 MHz, CDCl_3_): δ 7.64 (s, 1H), 6.79–6.77 (m, 2H), 6.37–6.35 (m, 2H), 3.72 (s, 3H), 3.65–3.57 (br m, 2H), 3.20–3.16 (br m, 2H), 2.33–2.28 (br m, 2H), 2.30 (s, 3H), 2.10–1.98 (br m, 2H). ^13^C NMR (100 MHz, CDCl_3_): δ 158.3, 140.6, 136.5, 122.3, 112.6, 112.4, 56.9, 35.5, 24.8, 23.1. MS (ESI): *m*/*z* 274 [M + H]^+^.

##### (4-Methylpiperazin-1-yl)[1-phenyl-5-(1*H*-pyrrol-1-yl)-1*H*-pyrazol-4-yl]methanone (**4**)

Prepared from the acid **59** and 1-methylpiperazine. Eluent: DCM/MeOH (97:3). Yield: 51%. Mp 156–158 °C. ^1^H NMR (400 MHz, CDCl_3_): δ 7.85 (s, 1H), 7.33 (s, 3H), 7.16–7.11 (m, 2H), 6.64 (s, 2H), 6.27 (s, 2H), 3.70 (br s, 2H), 3.23 (br s, 2H), 2.36 (br s, 2H), 2.22 (s, 3H), 2.04 (br s, 2H). ^13^C NMR (100 MHz, CDCl_3_): δ 162.1, 140.0, 137.8, 136.2, 136.2, 129.2, 128.2, 123.2, 121.8, 112.0, 111.4, 54.5, 45.9, 41.8. MS (ESI): *m*/*z* 336 [M + H]^+^.

##### 1-Phenyl-N-(piperidin-1-yl)-5-(1*H*-pyrrol-1-yl)-1*H*-pyrazole-4-carboxamide (**5**)

Obtained from the acid **59** and 1-aminopiperidine. Eluent: DCM/MeOH (95:5). Yield: 33%. Mp 165–167 °C. ^1^H NMR (400 MHz, CDCl_3_): δ 8.24 (s, 1H), 7.34–7.30 (m, 3H), 7.19–7.17 (m, 2H), 6.76 (s, 2H), 6.45 (s, 2H), 2.56–2.52 (m, 4H), 1.67–1.63 (m, 4H), 1.36–1.33 (m, 2H). ^13^C NMR (100 MHz, CDCl_3_): δ 158.2, 141.7, 137.6, 135.8, 129.2, 128.2, 122.5, 122.4, 113.9, 112.4, 56.9, 24.8, 23.1. MS (ESI): *m*/*z* 336 [M + H]^+^.

##### N-(4-Chlorophenyl)-1-phenyl-5-(1*H*-pyrrol-1-yl)-1*H*-pyrazole-4-carboxamide (**6**)

Prepared from the acid **59** and 4-chloroaniline. Yield: 38%. Mp 197–198 °C. ^1^H NMR (400 MHz, CDCl_3_): δ 8.31 (s, 1H), 7.35 (s, 3H), 7.26–7.22 (m, 4H), 6.87 (s, 2H), 6.79 (s, 2H), 6.55 (s, 2H). ^13^C NMR (100 MHz, CDCl_3_): δ 158.6, 141.8, 137.4, 136.3, 135.9, 129.4, 129.1, 128.9, 128.5, 122.8, 122.6, 120.8, 114.7, 113.0. MS (ESI): *m*/*z* 385 [M + Na]^+^.

##### [1-Phenyl-5-(1*H*-pyrrol-1-yl)-1*H*-pyrazol-4-yl][4-(pyrimidin-2-yl)piperazin-1-yl]methanone (**7**)

Prepared from the acid **59** and 1-(2-pyrimidyl)piperazine. Yield: 73%. Eluent DCM/MeOH (95:5). Mp 218–219 °C. ^1^H NMR (400 MHz,CDCl_3_): δ 8.28 (m, 2H), 7.91 (s, 1H), 7.34 (m, 3H), 7.14–7.13 (m, 2H), 6.66 (m, 2H), 6.51 (m,1H), 6.27 (s, 2H), 3.81–3.62 (m, 4H), 3.44–3.22 (m, 4H). ^13^C NMR (100 MHz, CDCl_3_): δ 157.8, 140.2, 136.4, 129.3, 128.3, 127.5, 123.2, 122.6, 122.4, 121.7, 116.2, 111.7, 110.4, 56.4, 43.3. MS (ESI): *m*/*z* 422 [M + Na]^+^.

##### N-(Adamantan-1-yl)-1-phenyl-5-(1*H*-pyrrol-1-yl)-1*H*-pyrazole-4-carboxamide (**8**)

Obtained through a reaction between acid **59** and 1-aminoadamantane. Yield: 73%. Mp 167–169 °C. ^1^H NMR (400 MHz, CD_3_COCD_3_): δ 7.29 (s, 1H), 6.63 (m, 3H), 6.50 (m, 2H), 6.25 (s, 2H), 6.65 (s, 2H), 4.54 (br s, 1H), 1.26 (s, 3H), 1.15 (s, 6H), 0.91 (s, 6H). ^13^C NMR (100 MHz, CDCl_3_): δ 159.8, 141.5, 137.7, 135.6, 129.2, 128.1, 122.4, 122.3, 115.8, 112.4, 51.9, 41.5, 36.3, 29.4. MS (ESI): *m*/*z* 409 [M + Na]^+^.

##### *tert*-Butyl (*S*)-3-Phenyl-2-[[[1-phenyl-5-(1*H*-pyrrol-1-yl)-1*H*-pyrazol-4-yl]carbonyl]amino]propanoate (**9**)

Prepared from the acid **59** and L-phenylalanine *tert*-butyl ester hydrochloride. Eluent: PE/EtOAc (3:1). Yield: 90%. Oil. ^1^H NMR (400 MHz,CDCl_3_): δ 8.19 (s, 1H), 7.33–7.16 (m, 6H), 7.09–7.00 (m, 4H), 6.64 (d, *J* = 1.9 Hz, 2H), 6.36 (d, *J* = 2.0 Hz, 2H), 5.82 (br s, 1H), 4.88–4.80 (m, 1H), 3.08–3.03 (m, 1H), 2.95–2.88 (m, 1H), 1.31 (s, 9H). ^13^C NMR (100 MHz, CDCl_3_): δ 170.3, 160.4, 141.5, 137.6, 136.6, 136.2, 129.4, 129.2, 128.4, 128.2, 126.9, 122.5, 122.3, 113.4, 112.4, 82.2, 53.6, 37.9, 27.9.. MS (ESI): *m*/*z* 479 [M + Na]^+^.

##### Ethyl 3-[[N-Benzil-N-[1-phenyl-5-(1*H*-pyrrol-1-yl)-1*H*-pyrazol-4-yl]carbonyl]amino]propanoate (**10**)

Obtained through a reaction between acid **59** and ethyl 3-(benzylamino)propionate. Eluent: PE/EtOAc (3:1). Yield: 65%. White solid. Mp 93–96 °C. ^1^H NMR (400 MHz, CDCl_3_): δ 7.87 and 7.79 (s, 1H overall, rotamers), 7.38–7.22 (m, 6H), 7.18–6.98 (m, 4H), 6.66 (s, 2H), 6.27 (s, 2H), 4.68 and 4.45 (br m, 2H overall, rotamers), 4.11 (q, *J* = 7.1 Hz, 2H), 3.61 and 3.43 (br m, 2H overall, rotamers), 2.60 and 2.22 (br m, 2H overall, rotamers), 1.24 (t, *J* = 7.1 Hz, 3H). ^13^C NMR (100 MHz, CDCl_3_): 171.7, 170.8 (1 C), 164.1, 139.0, 137.9, 136.9, 136.5, 129.2, 128.9, 128.1, 127.7, 126.9, 123.1, 122.1, 112.7, 112.1 (1 C), 111.3, 60.6, 53.1, 47.7 (1 C), 43.7, 41.6 (1 C), 32.8, 32.3 (1 C), 14.2. MS (ESI): *m*/*z* 442 [M + H]^+^.

##### Methyl [[[1-Phenyl-5-(1*H*-pyrrol-1-yl)-1*H*-pyrazol-4-yl]carbonyl]amino]acetate (**11**)

Prepared from acid **59** and glycine methyl ester hydrochloride. Eluent: PE/EtOAc (3:1 to 1:1). Yield: 70%. White needles. Mp 139–141 °C. ^1^H NMR (400 MHz, CDCl_3_): δ 8.15 (s, 1H), 7.25 (m, 3H), 7.05 (m, 2H), 6.71 (s, 2H), 6.36 (s, 2H), 5.79 (br s, 1H), 4.00 (d, 2H), 3.65 (s, 3H). ^13^C NMR (100 MHz, CDCl_3_): δ 169.9, 160.9, 141.5, 137.6, 136.7, 129.2, 128.3, 122.5, 122.4, 113.2, 112.5, 52.3, 41.1. MS (ESI): *m*/*z* 347 [M + Na]^+^.

##### Ethyl (*S*)-2-[[[1-Phenyl-5-(1*H*-pyrrol-1-yl)-1*H*-pyrazol-4-yl]carbonyl]amino]propanoate (**12**)

Obtained from acid **59** and L-alanine ethyl ester hydrochloride. Eluent PE/EtOAc (3:1). Yield: 60%. Amorphous white solid. Mp 90–92 °C. ^1^H NMR (400 MHz, CDCl_3_): δ 8.22 (s, 1H), 7.34–7.32 (m, 3H), 7.15 (dd, *J* = 7.6, 2.1 Hz, 2H), 6.79 (s, 2H), 6.45 (s, 2H), 5.77 (br d, 1H), 4.66–4.59 (m, 1H), 4.17 (qd, *J* = 7.2, 1.8 Hz, 2H), 1.31–1.23 (m, 6H). ^13^C NMR (100 MHz, CDCl_3_): δ 172.5, 160.3, 141.4, 137.6, 136.6, 129.2, 128.2, 122.5, 122.4, 113.7, 112.4, 61.4, 47.9, 18.2, 14.1. MS (ESI): *m*/*z* 352 [M + Na]^+^.

##### Ethyl (*S*)-3-Methyl-2-[[[1-phenyl-5-(1*H*-pyrrol-1-yl)-1*H*-pyrazol-4-yl]carbonyl]amino]butanoate (**13**)

Prepared from acid **59** and L-valine ethyl ester hydrochloride. Eluent PE/EtOAc (3:1). Yield: 53%. Yellow solid. Mp 75–78 °C. ^1^H NMR (400 MHz, CDCl_3_): δ 8.23 (s, 1H), 7.33–7.31 (m, 3H), 7.14 (dd, *J* = 7.6, 2.0, 2H), 6.83–6.82 (m, 2H), 6.45–6.44 (m, 1H), 5.78 (d, *J* = 8.4 Hz, 1H), 4.60 (dd, *J* = 8.6, 4.6 Hz, 1H), 4.16 (q, *J* = 7.1 Hz, 2H), 2.13–2.04 (m, 1H), 1.26 (t, *J* = 7.1 Hz, 3H), 0.85 (d, *J* = 6.9 Hz, 3H), 0.68 (d, *J* = 6.9 Hz, 3H). ^13^C NMR (100 MHz, CDCl_3_): δ 171.5, 160.8, 141.7, 137.8, 136.3, 129.2, 128.2, 122.5, 113.9, 112.6, 61.1, 56.8, 30.8, 19.0, 17.2, 14.2. MS (ESI): *m*/*z* 380 [2M + Na]^+^.

##### *tert*-Butyl (*S*)-3-Phenyl-2-[[[5-(1*H*-pyrrol-1-yl)-1-[6-(p-tolyl)pyridin-2-yl]-1*H*-pyrazol-4-yl]carbonyl]amino]propanoate (**14**)

Prepared from acid **60** and L-phenylalanine *tert*-butyl ester hydrochloride. Eluent: PE/EtOAc (2:1). Yield: 94%. Amorphous white solid. ^1^H NMR (400 MHz, CDCl_3_): δ 8.14 (s, 1H), 7.76 (t, *J* = 7.9 Hz, 1H), 7.58 (d, *J* = 7.8 Hz, 1H), 7.50–7.48 (m, 3H), 7.21–7.19 (m, 5H), 7.11 (d, *J* = 8.0 Hz, 2H), 7.01 (d, *J* = 7.7 Hz, 2H), 6.74 (d, *J* = 4.0 Hz, 2H), 6.33 (d, *J* = 3.9 Hz, 1H), 5.60 (d, *J* = 7.6 Hz, 1H), 4.74 (dd, *J* = 13.8, 7.1 Hz, 1H), 2.96 (dd, *J* = 14.0, 6.2 Hz, 1H), 2.80 (dd, *J* = 14.0, 7.1 Hz, 1H), 2.32 (s, 3H), 1.32 (s, 9H). ^13^C NMR (100 MHz, CDCl_3_): 170.3, 160.3, 156.3, 150.9, 141.9, 139.6, 139.2, 137.0, 136.3, 134.8, 129.4, 129.2, 128.4, 127.1, 126.8, 122.7, 118.8, 114.9, 114.8, 111.8. MS (ESI): *m*/*z* 547 [M + Na]^+^.

#### 4.1.6. General Procedure for the Synthesis of Acids **15**–**17**

A 2 M solution of LiOH (0.7 mL, 1.4 mmol) was added to a solution of the appropriate esters **11**–**13** (0.6 mmol) in THF (2 mL) and MeOH (1 mL). After stirring at room temperature for 2–4 h, the mixture was concentrated, diluted with water, and brought to a pH of 1–2 with conc. HCl. The solid was extracted with EtOAc and the combined organic layer was washed with brine and dried over anhydrous sodium sulfate. Evaporation of the solvent left a solid, which was purified by trituration with PE/Et_2_O.

##### [[[1-Phenyl-5-(1*H*-pyrrol-1-yl)-1*H*-pyrazol-4-yl]carbonyl]amino]acetic Acid (**15**)

Prepared through the hydrolysis of **11**. Yield: 62%. White solid. Mp 191–194 °C. ^1^H NMR (400 MHz, CD_3_COCD_3_): δ 10.94 (br s, 1H), 8.08 (s, 1H), 7.31–7.30 (m, 3H), 7.12–7.10 (m, 2H), 6.83 (s, 2H), 6.77 (br s, 1H), 6.19 (s, 2H), 3.95 (d, *J* = 5.5 Hz, 2H). ^13^C NMR (100 MHz, CD_3_OD): δ 171.4, 162.3, 139.8, 138.6, 137.6, 128.9, 128.3, 123.2, 122.6, 112.1, 110.8, 40.4. MS (ESI): *m*/*z* 309 [M − H]^−^.

##### (*S*)-2-[[[1-Phenyl-5-(1*H*-pyrrol-1-yl)-1*H*-pyrazol-4-yl]carbonyl]amino]propanoic Acid (**16**)

Obtained via the hydrolysis of **12**. Yield: 60%. Pale-pink solid. Mp 142–145 °C. ^1^H NMR (400 MHz, CDCl_3_): δ 9.43 (s, 1H), 8.23 (s, 1H), 7.33–7.32 (m, 3H), 7.17–7.15 (m, 2H), 6.77 (s, 2H), 6.43 (s, 1H), 5.78 (d, *J* = 6.7 Hz, 1H), 4.64–4.57 (m, 1H), 1.32 (d, *J* = 7.1 Hz, 3H). ^13^C NMR (100 MHz, CDCl_3_): δ 176.1, 161.0, 141.5, 137.4, 136.9, 129.3, 128.4, 122.6, 122.4, 113.4, 112.6, 48.0, 17.7. MS (ESI): *m*/*z* 324 [M − H]^−^.

##### (*S*)-3-Methyl-2-[[[1-phenyl-5-(1*H*-pyrrol-1-yl)-1*H*-pyrazol-4-yl]carbonyl]amino]butanoic Acid (**17**)

Prepared through the hydrolysis of **13**. Yield: 77%. Light-grey solid. Mp 150–152 °C. ^1^H NMR (400 MHz, CDCl_3_): δ 8.25 (s, 1H), 7.34–7.32 (m, 3H), 7.16–7.13 (m, 2H), 6.82 (s,2H), 6.45 (s,2H), 5.75 (d, *J* = 8.3 Hz, 1H), 4.59 (dd, *J* = 8.3, 4.5 Hz, 1H), 2.15 (dq, *J* = 13.6, 6.9 Hz, 1H), 0.88 (d, *J* = 6.8 Hz, 3H), 0.71 (d, *J* = 6.9 Hz, 3H). ^13^C NMR (100 MHz, CDCl_3_): δ 175.6, 161.3, 141.8, 137.5, 136.5, 129.3, 128.3, 122.5, 113.6, 112.8, 56.9, 30.2, 19.1, 17.0. MS (ESI): *m*/*z* 352 [M − H]^−^.

#### 4.1.7. N-[N-[[1-Phenyl-5-(1*H*-pyrrol-1-yl)-1*H*-pyrazol-4-yl]carbonyl]glycyl]-L-alanine Ethyl Ester (**18**)

Prepared from the acid **15** and L-alanine ethyl ester hydrochloride according to the general procedure used for the synthesis of amides **3**–**14**. Eluent: PE/EtOAc (1:1). Yield: 80%. White solid. Mp 116–117 °C. ^1^H NMR (400 MHz, CDCl_3_): δ 8.22 (s, 1H), 7.33–7.31 (m, 3H), 7.14–7.11 (m, 2H), 6.79–6.78 (m, 3H), 6.42 (s, 2H), 6.10 (br t, 1H), 4.58–4.51 (m, 1H), 4.19 (q, *J* = 7.1, 2H), 4.0 (d, *J* = 3.6, 2H), 2.33 (br s, 1H), 1.40 (d, *J* = 7.2, 3H), 1.27 (t, *J* = 7.1 3H). ^13^C NMR (100 MHz, CDCl_3_): δ 172.7, 168.2, 161.4, 141.2, 137.6, 137.1, 129.2, 128.3, 122.6, 122.5, 113.0, 112.4, 61.5, 48.2, 43.0, 18.3, 14.1. MS (ESI): *m*/*z* 432 [M + Na]^+^.

#### 4.1.8. N-[N-[[1-Phenyl-5-(1*H*-pyrrol-1-yl)-1*H*-pyrazol-4-yl]carbonyl]glycyl]-L-alanine (**19**)

The hydrolysis of **18**, according to the general procedure described for the synthesis of acids **15**–**17**, produced the title compound **19**, which was purified via recrystallization from MeOH/H_2_O. Yield: 81%. Light-yellow solid. Mp 80–84 °C. ^1^H NMR (400 MHz, CD_3_OD): δ 8.10 (s, 1H), 7.28–7.26 (m, 3H), 7.08–7.05 (m, 2H), 6.71–6.70 (m, 2H), 6.19–6.18 (m, 2H), 4.82 (br s, 2H), 4.35 (q, *J* = 7.3, 1H), 3.90 (q*, J* = 16.7, 2H), 1.32 (d, *J* = 7.3, 3H). ^13^C NMR (100 MHz, CD_3_OD): δ 174.4, 169.6, 162.3, 139.9, 138.7, 137.6, 128.9, 128.3, 123.2, 122.6, 112.2, 110.8, 48.0, 41.8, 16.4. MS (ESI): *m*/*z* 380 [M − H]^−^.

#### 4.1.9. General Procedure for the Synthesis of **61** and **62**

A solution of the amino derivatives **51** and **53** (3.6 mmol), DMAP (44 mg, 0.36 mmol), and di-tert-butyl dicarbonate (1.57 g, 7.2 mmol) in dry DCM (40 mL) was stirred at room temperature for 2–4 h. Afterward, it was washed with 1 N HCl, then brine, and finally, dried over anhydrous sodium sulfate. The removal of the solvent left an oily residue, which was purified by using flash chromatography on a silica gel eluted with PE/EtOAc (4:1) to produce the pure compounds **61** and **62**.

##### Ethyl 5-(*tert*-Butoxycarbonylamino)-1-phenyl-1*H*-pyrazole-4-carboxylate (**61**)

Prepared from **51**. Yield: 90%. White solid. Mp 91–93 °C. ^1^H NMR (400 MHz, CDCl_3_): δ 8.04 (s, 1H), 7.41–7.35 (m, 5H), 4.24 (q, 2H), 1.29–1.24 (superimposed signals, 12H). MS (ESI): *m*/*z* 332 [M + H]^+^.

##### Ethyl 5-(*tert*-Butoxycarbonylamino)-1-(4-nitrophenyl)-1*H*-pyrazole-4-carboxylate (**62**)

Prepared from **52**. Yield:79%. Pale-yellow solid. Mp 125–129 °C. ^1^H NMR (400 MHz, CDCl_3_): δ 8.25 (d, 2H), 8.02 (s, 1H), 7.61 (d, 2H), 4.20 (q, 2H), 1.20–1.30 (superimposed signals, 12H). MS (ESI): *m*/*z* 377 [M + H]^+^.

#### 4.1.10. General Procedure for the Synthesis of Acids **63** and **64**

To a solution of the esters **61** and **62** (4 mmol) in 50 mL of EtOH, 6 N KOH (27 mL, 0.16 mol) was added and the mixture was refluxed for 1 h. After cooling, the dark-orange solution was acidified with conc. HCl and the precipitate was extracted with DCM. The organic layer was washed with brine, dried (Na_2_SO4), and evaporated to leave a solid, which was sufficiently pure to be used in the next step.

##### 5-(*tert*-Butoxycarbonylamino)-1-phenyl-1*H*-pyrazole-4-carboxylic Acid (**63**)

Prepared from ester **61**. Yield: 73%. Mp 159–161 °C. ^1^H NMR (400 MHz, CDCl_3_): δ 7.99 (s, 1H), 7.53 (d, 2H), 7.43–7.20 (m, 3H), 1.19 (s, 9H). MS (ESI): *m*/*z* 302 [M − H]^−^.

##### 5-(*tert*-Butoxycarbonylamino)-1-(4-nitrophenyl)-1*H*-pyrazole-4-carboxylic Acid (**64**)

Obtained from ester **62**. Yield: 20%. Mp 212–213 °C. ^1^H NMR (400 MHz, CDCl_3_): δ 8.28 (d, 2H), 8.03 (s, 1H), 7.76 (d, 2H), 5.23 (s, 1H), 1.26 (s, 9H). MS (ESI): *m*/*z* 346 [M − H]^−^.

#### 4.1.11. General Procedure for the Synthesis of Amide Derivatives **20**–**22**

To a solution of the carboxylic acids **63** and **64** (1 mmol) in DCM (50 mL), HOBt (135 mg, 1 mmol), EDC (230 mg, 1.2 mmol), and the appropriate amine (1.5 mmol) were added successively. After stirring at room temperature for 2–4 h, the solution was washed with 1 N HCl, 10% NaHCO_3_ solution, and brine. After drying on sodium sulfate, the solvent was removed under reduced pressure and the residue was purified as described below.

##### N-(4-Bromobenzyl)-5-(*tert*-butoxycarbonylamino)-1-phenyl-1*H*-pyrazole-4-carboxamide (**20**)

Prepared from acid **63** and 4-bromobenzylamine. Purified via flash chromatography on a silica gel eluted with PE/EtOAc (1:1). Yield: 39%. White solid. Mp 175–176 °C. ^1^H NMR (400 MHz, CD_3_OD): δ 7.97 (s, 1H), 7.45–7.37 (m, 7H), 7.22 (d, *J* = 8.3 Hz, 2H), 4.43 (s, 2H), 1.20 (s, 9H). ^13^C NMR (100 MHz, CD_3_OD): δ 160.0, 153.6, 139.0, 138.4, 138.1, 137.0, 134.7, 131.2, 129.1, 128.9, 128.3, 124.2, 120.5, 81.2, 41.9, 26.9. MS (ESI): *m*/*z* 493 [M + Na]^+^.

##### N-(4-Bromobenzyl)-5-(*tert*-butoxycarbonylamino)-1-(4-nitrophenyl)-1*H*-pyrazole-4-carboxamide (**21**)

Obtained through a reaction between acid **64** and 4-bromobenzylamine. Purified via recrystallization from MeOH. Yield: 51%. White solid. Mp 169–171 °C. ^1^H NMR (400 MHz, DMSO-d6): δ 9.17 (br s, 1H), 8.62 (br t, 1H), 8.33 (d, *J* = 8.7 Hz, 2H), 8.16 (d, *J* = 2.7, 1H), 7.76 (d, *J* = 8.7 Hz, 2H), 7.44 (d, *J* = 8.2 Hz, 2H), 7.21 (d, *J* = 8.1 Hz, 2H), 4.34 (d, *J* = 4.2 Hz, 2H), 1.17 (s, 9H). ^13^C NMR (100 MHz, DMSO-d6): δ 161.6, 157.3, 146.5, 144.1, 140.7, 139.5, 138.4, 131.6, 130.0, 125.3, 124.2, 120.3, 112.9, 80.9, 41.9, 28.1. MS (ESI): *m*/*z* 538 [M + Na]^+^.

##### 5-(*tert*-Butoxycarbonylamino)-1-(4-nitrophenyl)-N-[[5-(4-trifluoromethoxyphenyl)pyridin-2-yl]methyl]-1*H*-pyrazole-4-carboxamide (**22**)

Prepared from acid **64** and [5-[4-(trifluoromethoxy)phenyl]pyridin-2-yl]methanamine [16]. The yellowish compound was purified through repeated triturations with EtOAc until a white solid was obtained. Yield: 59%. Mp 197–199 °C. ^1^H NMR (400 MHz, DMSO-d6): δ 9.22 (s, 1H), 8.76 (d, *J* = 7.0 Hz, 2H), 8.34 (d, *J* = 7.2 Hz, 2H), 8.22 (s, 1H), 8.00 (d, *J* = 8.2 Hz, 1H), 7.77 (d, *J* = 8.8 Hz, 3H), 7.46–7.36 (m, 2H), 4.51 (d, *J* = 5.6 Hz, 2H), 1.16 (s, 9H). ^13^C NMR (100 MHz, CDCl_3_): δ 163.1, 154.7, 151.6, 149.6, 146.3, 145.3, 141.0, 140.0, 138.6, 136.5, 135.4, 135.0, 128.6, 124.6, 122.9, 122.8, 121.7, 107.4, 82.6, 43.3, 27.9. MS (ESI): *m*/*z* 597 [M − H]^−^.

#### 4.1.12. General Procedure for the Synthesis of Amides **23**–**39**

These compounds were prepared according to the general procedure reported for the synthesis of amides **3**–**14** and **20**–**22**, and purified as reported below.

##### N-(Adamantan-3-ol-1-yl)-1-phenyl-5-(1*H*-pyrrol-1-yl)-1*H*-pyrazole-3-carboxamide (**23**)

Prepared from **65** and 3-aminoadamantan-1-ol. Purified through recrystallization from MeOH. Yield: 96%. Colorless crystals. Mp 217–221 °C. ^1^H NMR (400 MHz, CDCl_3_): δ 7.37–7.36 (m, 3H), 7.18–7.16 (m, 2H), 6.89 (s, 1H), 6.83 (s, 1H), 6.63–6.62 (m, 2H), 6.26–6.25 (m, 2H), 2.32 (s, 2H), 2.07 (q, *J* = 11.7, 4H), 1.91 (s, 2H), 1.75 (q, *J* = 11.6, 4H), 1.65–1.55 (m, 2H). ^13^C NMR (100 MHz, CDCl_3_): δ 160.4, 147.4, 140.6, 137.8, 129.3, 128.5, 123.5, 122.1, 110.9, 102.7, 69.1, 54.4, 49.1, 44.1, 40.4, 35.0, 30.7. MS (ESI): *m*/*z* 425 [M + Na] ^+^.

##### N-(Adamantan-1-yl)-4-cyano-1-phenyl-5-(1*H*-pyrrol-1-yl)-1*H*-pyrazole-3-carboxamide (**24**)

Prepared from **66** and 1-aminoadamantane. Purified through recrystallization from EtOH/DCM. Yield: 90%. White solid. Mp 227–229 °C. ^1^H NMR (400 MHz, CDCl_3_): δ 7.46–7.44 (m, 3H), 7.21–7.18 (m, 2H), 6.72–6.70 (m, 2H), 6.59 (br s, 1H), 6.34–6.32 (m, 2H), 2.16 (s, 9H), 1.73 (s, 6H). ^13^C NMR (100 MHz, CDCl3): δ 157.6, 148.2, 144.5, 136.7, 129.7, 123.9, 121.3, 112.4, 111.3, 88.8, 52.6, 41.6, 36.3, 29.5. MS (ESI): *m*/*z* 434 [M + Na]^+^.

##### *tert*-Butyl (*S*)-3-Phenyl-2-[[[4-cyano-1-phenyl-5-(1*H*-pyrrol-1-yl)-1*H*-pyrazol-3-yl]carbonyl]amino]propanoate (**25**)

Prepared from **66** and L-phenylalanine *tert*-butyl ester hydrochloride. Purified via flash chromatography on a silica gel, eluent PE/EtOAc (3:1), followed by trituration with PE/Et_2_O. Yield: 56%. White solid. Mp 93–94 °C. ^1^H NMR (400 MHz, CDCl_3_): δ 7.46–7.37 (m, 5H), 7.30–7.17 (m, 6H), 6.74–6.73 (m, 2H), 6.35–6.34 (m, 2H), 4.99 (dd, *J* = 14.1, 6.2 Hz, 1H), 3.24 (d, *J* = 6.1 Hz, 2H), 1.42 (s, 9H). ^13^C NMR (100 MHz, CDCl_3_): δ 170.3, 158.3, 146.7, 144.5, 136.7, 136.1, 129.7, 129.6, 129.5, 128.5, 127.0, 123.8, 121.4, 112.5, 111.0, 89.1, 82.7, 53.6,38.3, 28.0. MS (ESI): *m*/*z* 504 [M + Na]^+^.

##### (4-Ethoxycarbonylpiperazin-1-yl)[4-methyl-1-phenyl-5-(1*H*-pyrrol-1-yl)-1*H*-pyrazol-3-yl]methanone (**26**)

Prepared from **67** and ethyl 1-piperazinecarboxylate. Purified via flash chromatography on a silica gel, eluent PE/EtOAc (3:1). Yield: 81%. White solid. Mp 130–131 °C. ^1^H NMR (400 MHz, CDCl_3_): δ 7.41–7.21 (m, 3H), 7.06 (d, *J* = 8.2 Hz, 2H), 6.64 (s, 2H), 6.32 (s, 2H), 4.18 (q, *J* = 7.1 Hz, 2H), 3.97 (br s, 2H), 3.92 (br s, 2H), 3.61 (d, *J* = 4.2 Hz, 2H), 2.14 (s, 3H), 1.29 (t, *J* = 7.1 Hz, 3H). ^13^C NMR (100 MHz, CDCl_3_): δ 163.1, 155.5, 144.6, 138.0, 137.4, 129.2, 127.7, 122.2, 122.1, 115.1, 110.9, 61.4, 47.1, 44.3, 43.6, 42.2, 14.7, 7.9. MS (ESI): *m*/*z* 407 [M + Na]^+^.

##### N-(Adamantan-3-ol-1-yl)-4-methyl-1-phenyl-5-(1*H*-pyrrol-1-yl)-1*H*-pyrazole-3-carboxamide (**27**)

Prepared from **67** and 3-aminoadamantan-1-ol. Purified via flash chromatography on a silica gel, eluent PE/EtOAc (2:1 to 1:1), followed by trituration with PE/Et2O. Yield: 34%. White solid. Mp 137–138 °C. 1H NMR (400 MHz, CDCl3): δ 7.32–7.31 (m, 3H), 7.11–7.09 (d, *J* = 8.0, 2H), 6.90 (s, 1H), 6.61 (s, 2H), 6.30 (s, 2H), 2.32 (s, 2H), 2.27 (s, 2H), 2.16 (s, 2H), 2.08 (s, 3H), 1.79–1.71 (m, 4H), 1.66–1.55 (m, 4H). 13C NMR (100 MHz, CDCl3): δ 161.8, 144.0, 138.0, 137.9, 129.2, 127.9, 122.5, 122.3, 115.1, 110.8, 69.2, 54.3, 49.3, 44.2, 40.5, 35.0, 30.7, 8.2. MS (ESI): *m*/*z* 439 [M + Na]^+^, 417 [M + H]^+^.

##### [1-(4-Chlorophenyl)-4-methyl-5-(1*H*-pyrrol-1-yl)-1*H*-pyrazol-3-yl](4-methylpiperazin-1-yl)methanone (**28**)

Prepared from **68** and 1-methylpiperazine. Purified via flash chromatography on a silica gel eluted with EtOAc, followed by recrystallization from MeOH. Yield: 91%. White needles. Mp 178–182 °C. ^1^H NMR (400 MHz, CDCl_3_): δ 7.26 (d, *J* = 8.8 Hz, 2H), 6.97 (d, *J* = 8.8 Hz, 2H), 6.63 (s, 2H), 6.34 (s, 2H), 3.92–3.90 (m, 2H), 3.88–3.85 (m, 2H), 2.56–2.48 (m, 4H), 2.36 (s, 3H), 2.11 (s, 3H). ^13^C NMR (100 MHz, CDCl_3_): δ 162.7, 145.4, 137.1, 136.6, 133.2, 129.3, 123.1, 122.1, 114.9, 111.2, 55.6, 54.8, 47.1, 46.0, 42.1, 7.8. MS (ESI): *m*/*z* 384 [M + H]^+^.

##### N-(Benzo[*d*][1,3]dioxol-5-ylmethyl)-1-(4-chlorophenyl)-4-methyl-5-(1*H*-pyrrol-1-yl)-1*H*-pyrazole-3-carboxamide (**29**)

Prepared from **68** and piperonylamine. Purified via flash chromatography on a silica gel eluted with PE/EtOAc 4:1, followed by recrystallization from MeOH. Yield: 74%. White crystals. Mp 144–148 °C. ^1^H NMR (400 MHz, CDCl_3_): δ 7.30–7.26 (m, 3H), 6.99 (d, *J* = 7.8 Hz, 2H), 6.89 (d, *J* = 1.4 Hz, 1H), 6.84 (dd, *J* = 8.0, 1.4 Hz, 1H), 6.78 (d, *J* = 7.9 Hz, 1H), 6.62 (s, 2H), 6.34 (s, 2H), 5.95 (s, 2H), 4.56 (d, *J* = 6.0 Hz, 2H), 2.30 (s, 3H). ^13^C NMR (100 MHz, CDCl3): δ 162.1, 148.0, 147.0, 143.6, 137.9, 136.4, 133.6, 132.3, 129.3, 123.3, 122.2, 121.2, 115.8, 111.2, 108.5, 108.3, 101.1, 42.8, 8.1. MS (ESI): *m*/*z* 457 [M + Na]^+^.

##### [1-(4-Chlorophenyl)-4-methyl-5-(1*H*-pyrrol-1-yl)-1*H*-pyrazol-3-yl](morpholin-4-yl)methanone (**30**)

Prepared from **68** and morpholine. Purified via flash chromatography on a silica gel (eluent: EtOAc), followed by recrystallization from MeOH. Yield: 89%. White needles. Mp 174–177 °C. ^1^H NMR (400 MHz, CDCl_3_): δ 7.20–7.18 (m, 2H), 6.90–6.87 (m, 2H), 6.57–6.55 (m, 2H), 6.27–6.24 (m, 2H), 3.88–3.86 (m, 2H), 3.76–3.73 (m, 4H), 3.68–3.66 (m, 2H), 2.04 (s, 3H). ^13^C NMR (100 MHz, CDCl_3_): δ 162.7, 144.9, 137.2, 136.5, 133.3, 129.3, 123.1, 115.3, 111.2, 67.2, 66.9, 47.8, 42.6, 7.8. MS (ESI): *m*/*z* 393 [M + Na]^+^.

##### N-Benzyl-1-(4-chlorophenyl)-N-(2-hydroxyethyl)-4-methyl-5-(1*H*-pyrrol-1-yl)-1*H*-pyrazole-3-carboxamide (**31**)

Obtained through a reaction between **68** and N-benzylethanolamine. Purified via flash chromatography on a silica gel eluted with PE/EtOAc (1:1). Yield: 82%. Oil. Two rotamers: ^1^H NMR (400 MHz, CDCl_3_): δ 7.37–7.34 (m), 7.28 (d, *J* = 10.7 Hz), 7.19 (d, *J* = 7.5 Hz), 6.96 (d, *J* = 7.9 Hz), 6.82 (d, *J* = 7.5 Hz), 6.63 (d, *J* = 7.7 Hz, 2H), 6.35 (s), 5.04 (s), 4.88 (s), 4.42 (br s), 3.86 (s), 3.77–3.70 (m, 2H), 2.20 (s), 2.13 (s). ^13^C NMR (100 MHz, CDCl_3_): δ 166.2 and 164.3 (1 C), 145.2 and 145.1 (1 C), 137.7 and 137.3 (1 C), 137.2 and 137.0 (1 C), 136.5 and 136.1 (1 C), 133.7 and 133.2 (1 C), 129.6, 129.3, 128.7, 128.1, 127.6, 123.2, 122.8, 122.1, 115.9 and 115.7 (1 C), 111.4, 61.5, 59.6, 54.1, 49.5, 48.4, 7.9. MS (ESI): *m*/*z* 435 [M + H]^+^.

##### Ethyl 4-[[[1-(4-Chlorophenyl)-4-methyl-5-(1*H*-pyrrol-1-yl)-1*H*-pyrazol-3-yl]carbonyl]amino]benzoate (**32**)

Prepared through a reaction between **68** and ethyl 4-aminobenzoate. Purified via flash chromatography on a silica gel eluted with DCM, followed by recrystallization from EtOH. Yield: 70%. White needles. Mp 168–170 °C. ^1^H NMR (400 MHz, CDCl_3_): δ 8.98 (br s, 1H), 8.07 (d, *J* = 8.7 Hz, 2H), 7.80 (d, *J* = 8.7 Hz, 2H), 7.33 (d, *J* = 8.8 Hz, 2H), 7.06 (d, *J* = 8.8 Hz, 2H), 6.65–6.64 (m, 2H), 6.37–6.36 (m, 2H), 4.38 (q, *J* = 7.2 Hz, 2H), 2.34 (s, 3H), 1.41 (t, *J* = 7.1 Hz, 3H). ^13^C NMR (100 MHz, CDCl3): δ 166.2, 160.2, 143.3, 141.8, 138.4, 136.2, 134.0, 130.9, 129.5, 125.9, 123.5, 122.2, 118.8, 116.3, 111.4, 60.9, 14.4, 8.2. MS (ESI): *m*/*z* 449 [M + H]^+^.

##### 1-(4-Chlorophenyl)-4-methyl-N-(pyridin-4-ylmethyl)-5-(1*H*-pyrrol-1-yl)-1*H*-pyrazole-3-carboxamide (**33**)

Prepared through a reaction between **68** and 4-aminomethylpyridine. Purified via flash chromatography on a silica gel eluted with EtOAc, followed by recrystallization from PE/Et_2_O/DCM. Yield: 75%. White crystals. Mp 189–193 °C. ^1^H NMR (400 MHz, CDCl_3_): δ 8.60 (d, *J* = 5.0 Hz, 2H), 7.41 (t, *J* = 5.9 Hz, 1H), 7.37–7.31 (m, 4H), 7.01 (d, *J* = 8.9 Hz, 2H), 6.63–6.62 (m, 2H), 6.36–6.35 (m, 2H), 4.67 (d, *J* = 6.3 Hz, 2H) 2.30 (s, 3H). ^13^C NMR (100 MHz, CDCl3): δ 162.5, 149.1, 148.8, 143.1, 138.1, 136.3, 133.8, 129.4, 123.3, 122.6, 122.2, 115.9, 111.3, 41.8, 8.06. MS (ESI): *m*/*z* 392 [M + H]^+^.

##### N-Benzyl-1-(4-chlorophenyl)-N,4-dimethyl-5-(1*H*-pyrrol-1-yl)-1*H*-pyrazole-3-carboxamide (**34**)

Obtained through a reaction between **68** and N-benzylmethylamine. Purified via trituration with PE. Yield: 53%. White solid. Mp 104–108 °C. Two rotamers: ^1^H NMR (400 MHz, CDCl3): δ 7.46–7.13 (m), 6.99 (d, *J* = 7.4 Hz), 6.86 (d, *J* = 7.9 Hz), 6.63 (s), 6.35 (s), 4.95 (s), 4.81 (s), 3.25 (s), 3.08 (s), 2.15 (s). ^13^C NMR (100 MHz, CDCl3): δ 164.7 and 164.3 (1 C), 137.3 and 136.9 (1 C), 137.1 and 136.6 (1C), 133.2 and 133.1 (1 C), 129.3, 128.7, 128.2, 127.6, 127.4, 123.1, 122.8, 122.1, 115.2 and 115.1 (1 C), 111.2, 54.9 and 51.2 (1 C), 36.6 and 33.4 (1 C), 7.8. MS (ESI): *m*/*z* 405 [M + H]^+^.

##### 1-(4-Chlorophenyl)-4-cyano-N-(pyridin-4-ylmethyl)-5-(1*H*-pyrrol-1-yl)-1*H*-pyrazole-3-carboxamide (**35**)

Prepared through a reaction between **69** and 4-aminomethylpyridine. Purified via flash chromatography on a silica gel eluted with DCM/MeOH (99:1). Yield: 54%. Glassy solid. Mp 85–88 °C. ^1^H NMR (400 MHz, CDCl_3_): δ 8.59 (d, *J* = 5.4 Hz, 2H), 7.40 (d, *J* = 8.7 Hz, 2H), 7.34 (d, *J* = 5.3 Hz, 2H), 7.12 (d, *J* = 8.7 Hz, 2H), 6.73 (s, 2H), 6.38 (s, 2H), 4.70 (d, *J* = 6.3 Hz, 2H). ^13^C NMR (100 MHz, CDCl_3_): δ 158.9, 149.3, 147.8, 146.8, 144.6, 135.9, 135.0, 129.9, 124.9, 122.7, 121.3, 113.0, 110.6, 89.5, 42.1. MS (ESI): *m*/*z* 403 [M + H]^+^.

##### 1-(3,4-Dichlorophenyl)-N-(4-fluorobenzyl)-4-methyl-5-(1*H*-pyrrol-1-yl)-1*H*-pyrazole-3-carboxamide (**36**)

Prepared through a reaction between **70** and 4-fluorobenzylamine. Purified via flash chromatography on a silica gel eluted with PE/EtOAc (2:1). Yield: 77%. White solid. Mp 148–151 °C. ^1^H NMR (400 MHz, CDCl_3_): δ 7.51–7.29 (m, 4H), 7.25 (d, *J* = 2.3 Hz, 1H), 7.05 (t, *J* = 8.6, 2H), 6.73 (dd, *J* = 8.7, 2.3, 1H), 6.63 (s, 2H), 6.39 (s, 2H), 4.63 (d, *J* = 6.1, 2H), 2.30 (s, 3H)). ^13^C NMR (100 MHz, CDCl_3_): δ 163.4, 162.0, 161.0, 143.9, 137.9, 137.0, 134.3, 133.2, 131.7, 130.7, 129.6, 129.5, 123.8, 122.1, 120.4, 116.3, 115.6, 115.4, 111.7, 42.3, 8.1. MS (ESI): *m*/*z* 465 [M + Na]^+^.

##### 1-(3,4-Dichlorophenyl)-N-[4-(4-fluorophenyl)thiazol-2-yl]-4-methyl-5-(1*H*-pyrrol-1-yl)-1*H*-pyrazole-3-carboxamide (**37**)

Prepared through a reaction between **70** and 2-amino-4-(4-fluorophenyl)thiazole. Purified via flash chromatography on a silica gel eluted with PE/EtOAc (5:1). Yield: 20%. White solid. Mp 207–210 °C. ^1^H NMR (400 MHz, CDCl_3_): δ 10.54 (br s, 1H), 7.86–7.82 (m, 2H), 7.40 (d, *J* = 8.8 Hz, 1H), 7.27 (s, 1H), 7.15–7.08 (m, 3H), 6.83 (d, *J* = 8.7 Hz, 1H), 6.67 (s, 2H), 6.43 (s, 2H), 2.35 (s, 3H). ^13^C NMR (100 MHz, CDCl_3_): δ 163.9, 161.4, 159.4, 157.5, 149.0, 141.9, 138.3, 136.6, 133.4, 132.1, 130.8, 130.4, 127.8, 127.7, 123.6, 122.1, 120.4, 117.2, 115.7, 115.5, 111.9, 107.3, 7.9. MS (ESI): *m*/*z* 511 [M − H]^−^.

##### N-(Benzo[d][1,3]dioxol-5-ylmethyl)-1-(3,4-dichlorophenyl)-4-methyl-5-(2,5-dimethyl-1*H*-pyrrol-1-yl)-1*H*-pyrazole-3-carboxamide (**38**)

Prepared from **71** and piperonylamine. Purified via flash chromatography on a silica gel eluted with PE/EtOAc (2:1). Yield: 97%. Light-yellow solid. Mp 141–145 °C. ^1^H NMR (400 MHz, CDCl_3_): δ 7.46–7.30 (m, 4H), 7.22 (d *J* = 2.5 Hz, 1H), 6.92 (s, 1H), 6.88 (d, *J* = 7.9 Hz, 1H), 6.80 (d, *J* = 7.9 Hz, 1H), 6.82 (dd, *J* = 8.8, 2.5 Hz, 1H), 5.97 (d, *J* = 9.6 Hz, 2H), 4.58 (d, *J* = 6.0 Hz, 2H), 2.25 (s, 3H), 1.87 (s, 6H). ^13^C NMR (100 MHz, CDCl_3_): δ 162.0, 148.0, 147.0, 144.0, 137.4, 135.7, 133.4, 132.2, 131.2, 131.0, 128.9, 122.9, 121.2, 119.4, 118.5, 108.6, 108.3, 101.1, 42.8, 12.2, 8.3. MS (ESI): *m*/*z* 498 [M + H]^+^.

##### *tert*-Butyl (*S*)-3-Phenyl-2-[[[4-methyl-1-(2,4,6-trichlorophenyl)-5-(2,5-dimethyl-1*H*-pyrrol-1-yl)-1*H*-pyrazol-3-yl]carbonyl]amino]propanoate (**39**)

Prepared from **72** and L-phenylalanine *tert*-butyl ester hydrochloride. Purified via flash chromatography on a silica gel eluted with DCM. Yield: 58%. Glassy solid. Mixture of rotamers. Mp 72–81 °C. ^1^H NMR (400 MHz, CDCl_3_): δ 7.41–7.31 (m, 3H), 7.22–7.17 (m, 5H), 5.79–5.76 (m, 2H), 4.93–4.86 (m, 1H), 3.14 (t, *J* = 6.4 Hz, 2H), 2.09 and 2.07 (s, 3H overall), 1.94 and 1.92 (s, 3H overall), 1.89 and 1.87 (s, 3H overall), 1.36 and 1.34 (s, 9H overall). ^13^C NMR (100 MHz, CDCl_3_): δ 170.4, 161.6, 144.4, 138.5, 136.4, 135.4, 134.9, 132.8, 130.5, 130.3, 129.7, 129.6, 129.2, 129.1, 128.4, 126.9, 117.3, 108.1, 82.2, 53.3, 38.5, 28.0, 12.8, 12.7, 8.7. MS (ESI): *m*/*z* 639 [M + H]^+^.

#### 4.1.13. 4-[[[1-(4-Chlorophenyl)-4-methyl-5-(1*H*-pyrrol-1-yl)-1*H*-pyrazol-3-yl]carbonyl]amino]benzoic Acid (**40**)

A mixture of 32 (176 mg, 0.4 mmol) in MeOH (20 mL) and NaOH (208 mg, 0.52 mmol) in water (20 mL) was refluxed for 3 h. After cooling to room temperature, the solution was brought to a pH of 1–2 with conc. HCl. The precipitate was filtered under suction and washed thoroughly with water and then PE. The solid product was recrystallized with DMF to give the title compound (69 mg, 41% yield) as a white solid. Mp > 250 °C. ^1^H NMR (400 MHz, DMSO-d6): δ 12.67 (s, 1H), 10.42 (s, 1H), 7.90 (d, *J* = 8.7 Hz, 2H), 7.86 (d, *J* = 8.7 Hz, 2H), 7.42 (d, *J* = 8.6 Hz, 2H), 7.17 (d, *J* = 8.6 Hz, 2H), 6.86 (s, 2H), 6.23 (s, 2H), 2.09 (s, 3H).^13^C NMR (100 MHz, DMSO-d6): δ 167.4, 161.2, 143.9, 143.1, 138.5, 136.9, 133.2, 130.7, 129.6, 126.1, 125.0, 123.3, 120.1, 115.6, 111.3, 8.3. MS (ESI): *m*/*z* 421 [M + H]^+^. 

#### 4.1.14. General Procedure for the Synthesis of Amides **41**, **43**, and **48**

These compounds were prepared according to the general procedure reported for the synthesis of amides **23**–**39** and purified as reported below.

##### Methyl 3-[[[1-(4-Chlorophenyl)-4-methyl-5-(1*H*-pyrrol-1-yl)-1*H*-pyrazol-3-yl]carbonyl]amino]benzoate (**41**)

Prepared through a reaction between **68** and methyl 3-aminobenzoate. Purified via flash chromatography on a silica gel eluted with DCM/PE (1:1), followed by recrystallization from MeOH. Yield: 55%. White crystals. Mp 158–160 °C. ^1^H NMR (400 MHz, CDCl_3_): δ 8.93 (s, 1H), 8.23 (s, 1H), 8.10 (d, *J* = 7.6 Hz, 1H), 7.83 (d, *J* = 7.0 Hz, 1H), 7.47 (t, *J* = 6.6 Hz, 1H), 7.33 (d, *J* = 6.6 Hz, 2H), 7.07 (d, *J* = 6.7 Hz, 2H), 6.65 (s, 2H), 6.36 (s, 2H), 3.94 (s, 3H), 2.34 (s, 3H). MS (ESI): *m*/*z* 457 [M + Na]^+^. 

##### Methyl 2-[[[1-(4-Chlorophenyl)-4-methyl-5-(1*H*-pyrrol-1-yl)-1*H*-pyrazol-3-yl]carbonyl]amino]benzoate (**43**)

Prepared through a reaction between **68** and methyl 2-aminobenzoate. Purified via trituration with Et_2_O/PE. Yield: 53%. White solid. Mp 217–219 °C. ^1^H NMR (400 MHz, CDCl_3_): δ 12.36 (s, 1H), 8.87 (dd, *J* = 8.5, 1.0 Hz, 1H), 8.06 (dd, *J* = 8.0, 3.7 Hz, 1H), 7.57 (ddd, *J* = 8.6, 7.4, 1.5 Hz, 1H), 7.31–7.29 (m, 2H), 7.11–7.09 (m, 3H), 6.64 (dd, *J* = 5.0, 2.9 Hz, 2H), 6.35 (dd, *J* = 5.0, 2.8 Hz, 2H), 3.94 (s, 3H), 2.30 (s, 3H). MS (ESI): *m*/*z* 457 [M + Na]^+^. 

##### 1-(4-Chlorophenyl)-N-(4-methylthiophenyl)-4-methyl-5-(1*H*-pyrrol-1-yl)-1*H*-pyrazole-3-carboxamide (**48**)

Prepared through a reaction between **68** and 4-(methylthio)aniline. Purified via trituration with Et_2_O/PE. Yield: 94%. Beige solid. Mp 102–105 °C. ^1^H NMR (400 MHz, CDCl_3_): δ 8.76 (s, 1H), 7.63 (d, *J* = 8.7 Hz, 2H), 7.33–7.28 (m, 4H), 7.10–7.07 (m, 2H), 6.63–6.62 (m, 2H), 6.34–6.32 (m, 2H), 2.47 (s, 3H), 2.30 (s, 3H). MS (ESI): *m*/*z* 445 [M + Na]^+^. 

#### 4.1.15. General Procedure for the Synthesis of Acids **42** and **44**

These acids were prepared according to the same procedure used for the synthesis of **40**.

##### 3-[[[1-(4-Chlorophenyl)-4-methyl-5-(1*H*-pyrrol-1-yl)-1*H*-pyrazol-3-yl]carbonyl]amino]benzoic Acid (**42**)

Obtained from **41**. Purified via recrystallization from DMF. Yield: 82%. White crystals. Mp 248–250 °C. ^1^H NMR (400 MHz, CDCl_3_): δ 10.39 (s, 1H), 8.49–8.47 (m, 1H), 8.02 (ddd, *J* = 8.1, 2.1, 1.0 Hz, 1H), 7.66–7.64 (m, 1H), 7.47–7.43 (m, 3H), 7.23–7.20 (m, 2H), 6.91–6.89 (m, 2H), 6.28–6.27 (m, 2H), 2.13 (s, 3H). ^13^C NMR (100 MHz, DMSO-*d*_6_): δ 167.66, 161.09, 143.88, 139.25, 138.38, 136.88, 133.03, 131.74, 129.59, 129.18, 124.92, 124.72 (x2), 123.28, 121.62, 115.31, 111.53. MS (ESI): *m*/*z* 443 [M + Na]^+^.

##### 2-[[[1-(4-Chlorophenyl)-4-methyl-5-(1H-pyrrol-1-yl)-1H-pyrazol-3-yl]carbonyl]amino]benzoic Acid (**44**)

Prepared from **43.** No further purification was required. Yield: 61%. White solid. Mp 270–272 °C. ^1^H NMR (400 MHz, DMSO-*d*_6_): δ 12.58 (s, 1H), 8.76 (d, *J* = 8.2 Hz, 1H), 8.02 (dd, *J* = 7.9, 1.4 Hz, 1H), 7.62–7.60 (m, 1H) 7.45 (d, *J* = 8.8 Hz, 2H), 7.16–7.14 (m, 1H), 7.11 (d, *J* = 8.8, 2H), 6.92–6.91 (m, 2H), 6.28–6.27 (m, 2H), 2.15 (s, 3H). ^13^C NMR (100 MHz, DMSO-*d*_6_): δ 169.49, 160.72, 143.63, 141.30, 138.64, 136.56, 134.68, 133.03, 131.60, 129.66 (x2), 124.12, 123.24, 120.03, 116.68, 115.86, 111.33, 8.33. MS (ESI): *m*/*z* 443 [M + Na]^+^.

#### 4.1.16. 1-(4-Chlorophenyl)-N-(4-hydroxyphenyl)-4-methyl-5-(1*H*-pyrrol-1-yl)-1*H*-pyrazole-3-carboxamide (**45**)

To a solution of the carboxylic acid **68** (0.10 g, 0.33 mmol) in dry DMF (5 mL), PyBOP (0.26 g, 0.50 mmol), HOBt (40 mg, 0.33 mmol), and 4-aminophenol (40 mg, 0.36 mmol) were added successively. After stirring at room temperature for 4 h, the solution was washed with a 5% LiCl solution and brine. After drying on sodium sulfate, the solvent was removed under reduced pressure and the residue was purified through flash chromatography on a silica gel eluted with PE/AcOEt (2:1) to give 58 mg (45%) of the title compound as a beige solid. Mp 226–228 °C. ^1^H NMR (400 MHz, DMSO-*d*_6_): δ 9.94 (s, 1H), 9.21 (s, 1H), 7.53 (d, 2H), 7.46–7.44 (m, 2H), 7.19–7.17 (m, 2H), 6.89 (t, 2H), 6.70 (d, 2H), 6.27–6.26 (m, 2H), 2.10 (s, 3H). ^13^C NMR (100 MHz, DMSO-*d*_6_): δ 160.41, 154.19, 144.42, 138.23 136.93 132.92, 130.49, 129.47 (x2), 124.76, 123.27, 122.57, 115.23, 111.21, 8.01. MS (ESI): *m*/*z* 415 [M + Na]^+^.

#### 4.1.17. 1-(4-Chlorophenyl)-4-methyl-5-(1*H*-pyrrol-1-yl)-N-(4-sulfamoylphenyl)-1*H*-pyrazole-3-carboxamide (**46**)

Oxalyl chloride (60 µL, 0.668 mmol) and a drop of dry DMF were added to a solution of **68** (0.18 g, 0.608 mmol) in dry DCM (15 mL), which was cooled to 0 °C. After stirring for 1 h at room temperature, volatiles were evaporated and the solid residue was taken up in dry THF (10 mL). When the solution was cooled to 0 °C, sulfanilamide (0.10 g, 0.608 mmol) and TEA (0.13 mL, 0.91 mmol) were added and the mixture was stirred at room temperature for 2 h. The solvent was evaporated, and the residue was redissolved in DCM and washed with saturated NaHCO_3_ solution and brine. After drying on sodium sulfate, the solution was evaporated to leave a solid residue, which was triturated with PE/AcOEt to give 0.211 g (76%) of 46 as a white solid. Mp 238–239 °C. ^1^H NMR (400 MHz, DMSO-*d*_6_): δ 10.50 (s, 1H), 7.98 (d, *J* = 8.8, 2H), 7.77 (d, *J* = 8.8, 2H), 7.50–7.46 (m, 2H), 7.22 (dd, *J* = 11.0, 4.0 Hz, 4H), 6.90 (t, *J* = 2.1, 2H), 6.28–6.27 (m, 2H), 2.13 (s, 3H). ^13^C NMR (100 MHz, DMSO-*d*_6_): δ 161.23, 143.51, 141.79, 139.23, 138.40, 136.81, 132.96, 129.43 (x2), 126.93, 125.00, 123.30, 120.38, 115.54, 110.94. MS (ESI): *m*/*z* 478 [M + Na]^+^.

#### 4.1.18. Ethyl [[4-[1-(4-Chlorophenyl)-4-methyl-5-(1*H*-pyrrol-1-yl)-1*H*-pyrazole-3-carboxamido]phenyl]sulfonyl]carbamate (**47**)

To a suspension of **46** (50 mg, 0.11 mmol) in dry DCM (10 mL), a solution of ethyl chloroformate (0.02 mL, 0.22 mmol) and TEA (0.03 mL, 0.22 mmol) in 5 mL of DCM was added dropwise. After stirring for 1 h at room temperature, the mixture was diluted with further DCM and washed with an aqueous 10% citric acid solution, then brine, and dried over sodium sulfate. The removal of the solvent produced a residue, which was purified through flash chromatography on a silica gel eluted with PE/AcOEt (2:1 to 1:1) to provide 38 mg (65%) of 47, which was recrystallized from AcOEt (white solid). Mp 188–190 °C. ^1^H NMR (400 MHz, acetone-*d*_6_): δ 10.00 (s, 1H), 8.11 (d, J= 8.8, 2H), 7.98 (d, J= 8.8, 2H), 7.42 (d, J= 8.8, 2H), 7.19 (d, J= 8.8, 2H), 6.31 (d, *J* = 1.8, 2H), 2.22 (s, 3H). ^13^C NMR (100 MHz, acetone-*d*_6_): δ 205.25, 160.64, 150.97, 143.95, 143.08, 138.19, 136.80, 133.89, 133.07, 129.18, 124.09, 122.50, 116.26, 115.74, 110.90, 61.82, 13.49, 7.01. MS (ESI): *m*/*z* 550 [M + Na]^+^.

#### 4.1.19. 1-(4-Chlorophenyl)-4-methyl-N-[4-(S-methylsulfonimidoyl)phenyl]-5-(1*H*-pyrrol-1-yl)-1*H*-pyrazole-3-carboxamide (**49**)

To a solution of **48** (70 mg, 0.16 mmol) in MeOH (15 mL), an aqueous 25% ammonia solution (9 µL, 0.24 mmol) and (diacetoxyiodo)benzene (0.12 g, 0.38 mmol) were added, and after 2 h, the volatiles were removed under reduced pressure. The residue was dried in vacuo and triturated with PE/AcOEt to give 66 mg (88%) of compound **48** as a brown solid. Mp 207–210 °C. ^1^H NMR (400 MHz, CDCl_3_): δ 9.13 (s, 1H), 8.01 (d, 2H), 7.94 (d, 2H), 7.30 (d, 2H), 7.05 (d, 2H), 6.61 (s, 2H), 6.33 (s, 2H), 3.27 (s, 3H), 2.29 (s, 3H). ^13^C NMR (100 MHz (CDCl_3_): δ 159.91, 142.73, 138.48, 136.10, 135.11, 134.01, 129.30 (x2), 123.50, 122.15 (x2), 119.75, 116.39, 111.30, 46.16, 7.58. MS (ESI): *m*/*z* 454 [M + Na]^+^.

### 4.2. Antibacterial Susceptibility Testing

#### 4.2.1. Agar Diffusion-Based Methods

The compounds were provided as powders and dissolved in 100% DMSO at a final concentration of 50 mg/mL. When a compound was not soluble, DMSO was further added to lower the concentration to 25 mg/mL or, when necessary, 12.5 or 6.25 mg/mL, until the compound was completely solubilized. The primary screen used in the evaluation of the direct antibacterial activity through an agar diffusion test was implemented on a 8-channel, automated liquid-handling platform (PerkinElmer Janus Integrator, Waltham, Mass.), which was located under a custom-made class 1000 laminar flow cabinet (Faster, Cornaredo, Italy). Briefly, 1-well plates (Thermo Fisher Scientific, Waltham, Mass.), previously filled with Mueller–Hinton Agar medium, were inoculated with a suspension of the bacterium in a sterile 0.9% NaCl solution containing 1.5 × 10^8^ CFU/mL, using a sterile swab. Indicator organisms included 4 Gram-positive (*Bacillus subtilis* ATCC 6633, *Enterococcus faecalis* ATCC 29212, *Staphylococcus epidermidis* ATCC 14990, and *Streptococcus pyogenes* ATCC 12344) and 4 Gram-negative (*Escherichia coli* CCUG^T^, *Klebsiella pneumoniae* ATCC 13833, *Pseudomonas aeruginosa* ATCC 27853, and *Acinetobacter baumannii* ATCC 17978) strains, which were routinely propagated using Mueller–Hinton Agar plates. In total, 2 μL of each solution of compounds was dispensed on the plate surface (corresponding to 100, 50, 25, or 12.5 μg of compound according to its solubility). The controls included the solvent (100% DMSO), kanamycin, piperacillin, ceftazidime, vancomycin, and teicoplanin. The results were recorded as the diameter of the growth inhibition zone. The results were recorded as the diameter of the growth inhibition zone. The inhibition zones of the controls (max variation from expected diameter, ± 1 mm) were used as the validation criteria. The assays were performed at least in duplicate, and the position in which a single compound was spotted on the plate was randomly modified in any successive experiment.

#### 4.2.2. Broth Microdilution Methods and MIC Determinations

A rapid test to identify compounds showing potential synergistic activity with specific antibiotics was implemented in a microplate format (final volume of 100 μL). The compounds were tested at a fixed concentration of 64 µg/mL in Mueller–Hinton broth (residual DMSO concentration ≤0.07%, except for compounds **12**, **28**, and **36**, for which the residual DMSO concentration was 1%) in the absence and presence of a subinhibitory concentration (0.5 × MIC) of colistin (fixed concentration of 0.12 μg/mL for *Escherichia coli* CCUG^T^ and *Klebsiella pneumoniae* ATCC 13833, and of 0.25 μg/mL for *Acinetobacter baumannii* ATCC 17978 and *Pseudomonas aeruginosa* ATCC 27853). Briefly, 10-fold concentrated (640 µg/mL) solutions of each compound were prepared in Mueller–Hinton broth and diluted in 80 μL of either unsupplemented or colistin-supplemented Mueller–Hinton II broth (Becton Dickinson, Eysins, Switzerland) in the microplate well, prior to the addition of the bacterial inoculum (5 × 10^5^ CFU/mL, by adding 10 μL of a 5 × 10^6^ CFU/mL bacterial suspension prepared extemporaneously in Mueller–Hinton broth). After incubation for 18–24 h at 35 ± 2 °C, plates were inspected for growth inhibition.

The minimum inhibitory concentration (MIC) and minimum bactericidal concentration (MBC) of the compounds were determined in triplicate using Mueller–Hinton broth and a bacterial inoculum of 5 × 10^5^ CFU/mL, as recommended by the CLSI [26]. The minimum antibacterial concentration (MAC) of the compounds was determined in the presence of a fixed and subinhibitory concentration of colistin (as mentioned above) [21]. 

Carbapenem-resistant, colistin-resistant, and XDR clinical isolates were kindly provided by Prof. Gian Maria Rossolini (University of Florence, Italy). *Klebsiella pneumoniae* SI-004Bo is a KPC-producing, pandrug-resistant isolate (KKBO-4 [17]) selected in vivo during colistin therapy (colistin resistance mechanism, IS-mediated insertional inactivation of *mgrB*). *Acinetobacter baumannii* N50 is an OXA-24-producing clinical isolate resistant to all β-lactams, aminoglycosides, and fluoroquinolones [18].

#### 4.2.3. Chequerboard Analysis and FIC Determinations

The chequerboard analysis is a two-dimensional array in which individual microplate wells contain a unique combination of the concentrations of the tested compound and colistin. Tested concentrations of the compound ranged from 64 to 0.06 μg/mL along the x-axis, whereas polymyxin E1 concentrations ranged from 2 to 0.03 μg/mL (or 32–0.5 μg/mL when polymyxin E1-resistant clinical isolates were used) and varied along the y-axis. Determining the fractional inhibitory concentration (FIC) of both compounds and computing the average FIC index allowed us to determine whether a synergistic, additive, or antagonistic activity between the tested compounds could be observed. A synergistic activity occurred when the average FIC index was equal to or less than 0.5 [21,27].

#### 4.2.4. Kill-Curve Analysis

The time-dependent bacterial killing of colistin, compound **9**, or a combination thereof was investigated using established methods [25]. Briefly, a culture medium containing a starting inoculum of 5.0 × 10^6^ CFU/mL of *Acinetobacter baumannii* N50 clinical isolate was incubated aerobically at 35 °C under orbital agitation (200 rpm) in Mueller–Hinton broth, in the absence (growth control) or presence of either 2 μg/mL of colistin (a concentration equivalent to the susceptibility breakpoint), 8 µg/mL of compound **9**, or both. The bacterial count (expressed in CFU/mL) in the control and antibiotic-containing media was determined every hour for up to 6 h of incubation via serial 10-fold dilution and direct plating of 0.1 mL of the sample on the Mueller–Hinton Agar medium (without antibiotic), which was incubated for 24 h at 35 °C.

### 4.3. Cytotoxicity Assays

#### 4.3.1. Membrane Integrity Assays

The potential cytotoxic activity of compounds was evaluated using the commercially available integrity assay (CytoTox 96^®^ non-radioactive cytotoxicity assay, Promega). The compounds were tested for their ability to induce the lysis of HeLa cells by measuring the release of lactate dehydrogenase (LDH) after incubating the HeLa cell cultures (20,000 cells/well) for 24 h (37 °C, 5% CO_2_) in DMEM (Dulbecco’s Modified Eagle Medium) supplemented with 10% fetal bovine serum, 4.5 mg/mL glucose, and 2 mM L-glutamine in the absence and presence of varying concentrations of the compounds (up to 256 µg/mL). Further controls included samples containing the medium only or in which cell lysis was induced by the addition of 9% Triton X-100 (maximum LDH release control). The percentage of cytotoxicity was computed as 100 × (sample LDH release)/(maximum LDH release). The variation of the percentage of cytotoxicity allowed us to compute an IC_50_ value (compound concentration inducing 50% cytotoxicity).

#### 4.3.2. Cell Viability and Proliferation Assays

The cytotoxicity of the compounds was also evaluated by measuring the number of viable cells in the culture with respect to the control culture (cells treated with DMSO only) using the RealTime-Glo™ MT Cell Viability Assay in the presence of varying concentrations of the compound. The assay is a nonlytic homogeneous, bioluminescent method that can be used to measure cell viability/proliferation in real time using NanoLuc^®^ luciferase and a cell-permeant prosubstrate (MT Cell Viability Substrate). The potential cytotoxicity was tested by incubating Hela cells (1500 cells/well) in DMEM and in the presence of up to 128 µg/mL of the compound for up to 48 h.

#### 4.3.3. Hemolytic Activity

The hemolytic activity of compounds **1**, **2**, **8**, **9**, **45**, and **49** (0.025–10 μg) was investigated using the method described by Bechlars et al. [28]. Triton X-100 was used as the hemolysis control and DMSO as the negative control.

## Figures and Tables

**Table 1:** Identification of compounds exhibiting synergistic activity on Gram-negative bacteria (Eco, *Escherichia coli* CCUG^T^; Kpn, *Klebsiella pneumoniae* ATCC 13833; Pae, *Pseudomonas aeruginosa* ATCC 27853; Aba, *Acinetobacter baumannii* ATCC 17978) with colistin (0.5 × MIC). A green square indicates the absence of growth under these conditions.

Cpd *^a^*	Synergistic Activity (Growth Inhibition) *^b^*			
	Eco	Kpn	Pae	Aba
**3**				
**4**				
**5**				
**6**				
**7**				
**8**	■			■
**9**	■	■		■
**10**				
**11**			- *^c^*	
**12**				
**13**	■	■		
**14**				
**15**			-	
**16**				
**17**				
**18**	-		-	
**19**	-		-	
**20**		■		■
**21**	-	-	-	
**22**		■		■
**23**				
**24**				
**25**				
**26**				
**27**	■	■		
**28**	■	■		■
**29**				
**30**				
**31**	■	■		■
**32**	-	-	-	
**33**	-	-	-	
**34**	-	-	-	
**35**				
**36**				
**37**				
**38**				
**39**				■
**40**				
**42**				
**44**				
**45**	■	■		■
**46**				
**47**				
**49**	■	■		■

*^a^*Compounds tested at 64 µg/mL, 1% DMSO was used as the negative control (no growth inhibition observed); *^b^*colistin concentration was 0.12 µg/mL (Eco and Kpn) or 0.25 µg/mL (Pae and Aba), corresponding to 0.5 × MIC; *^c^*-, not determined due to limited solubility of the compound in these conditions.

**Table 2:** MAC (minimum antibacterial concentration) values of selected compounds on representative Gram-negative bacteria (Eco, *Escherichia coli* CCUG^T^; Kpn, *Klebsiella pneumoniae* ATCC 13833; Aba, *Acinetobacter baumannii* ATCC 17978), when tested in the presence of 0.5 × MIC colistin. The minimal ratio between the MIC of the compound alone and the MAC in the presence of colistin is given between parentheses and used to quantify the improvement of the antibacterial activity of the tested compounds when tested in the presence of colistin. Data for parent compounds **1** and **2** are provided for comparison [12].

Compound	MAC (µg/mL) (MIC/MAC Ratio)		
	Eco	Kpn	Aba
**1**	64 (8)	64 (8)	32 (16)
**8**	32 (16)	- *^a^*	4 (128)
**9**	8 (64)	8 (64)	4 (128)
**13**	64 (8)	32 (16)	-
**20**	-	64 (8)	64 (8)
**22**	-	64 (8)	64 (8)
**2**	64 (8)	32 (16)	16 (32)
**27**	32 (16)	32 (16)	-
**28**	64 (8)	64 (8)	64 (8)
**31**	32 (16)	16 (32)	32 (16)
**39**	-	-	32 (16)
**45**	4 (128)	4 (128)	8 (64)
**49**	8 (64)	4 (128)	8 (64)

*^a^*No synergistic activity.

**Table 3:** Chequerboard analysis and average FIC index (FICI) values determined via the combination of colistin and pyrazolo-4-carboxamides **1**, **8**, and **9** and pyrazolo-3-carboxamides **2**, **45**, and **49** on *E. coli*, *K. pneumoniae*, and *A. baumannii* strains, including colistin-resistant clinical isolates.

Bacterial Strain	Average FIC Index					
	1	8	9	2	45	49
*E. coli* CCUG^T^	0.5	0.38	0.33	0.31	0.31	0.32
*K. pneumoniae* ATCC 13833	0.5	0.5	0.32	0.5	0.31	0.39
*K. pneumoniae* SI-004Bo *^a^*	1	1	1	1	1	1
*A. baumannii* ATCC 17978	0.38	0.31	0.34	0.39	0.31	0.38
*A. baumannii* N50 *^b^*	0.5	0.24	0.23	0.4	0.26	0.31

*^a^ Klebsiella pneumoniae* exhibiting a pandrug-resistant phenotype (colistin MIC, 64 μg/mL) [17]. *^b^*A multidrug-resistant (MDR) *Acinetobacter baumannii* clinical isolate (colistin MIC, 32 μg/mL) [18].

**Table 4:** Cytotoxicity (membrane-damaging activity) of compounds **8** and **9**, tested alone or in the presence of 512 µg/mL of colistin.

Compound	IC_50_ (μg/mL)
Colistin	>256
**8**	>256
**8** + colistin	16.4
**9**	>256
**9** + colistin	>256
